# PKCγ-mediated Phosphorylation of Mtss1 Regulates the Dendritic Outgrowth and Spine Development of Cerebellar Purkinje Cells

**DOI:** 10.1007/s12035-025-05526-9

**Published:** 2025-11-25

**Authors:** Paula Torrents-Solé, Zsófia Sziber, Etsuko Shimobayashi, Josef P. Kapfhammer

**Affiliations:** 1 Anatomical Institute, Department of Biomedicine, University of Basel, CH-4056 Basel, Switzerland; 2 Present Address: Takeda Pharmaceuticals, Yokohama, Japan

**Keywords:** Purkinje cells, Spinocerebellar ataxia, PKCγ, Morphology, Dendritic arbours, Spines, Neurodevelopment

## Abstract

Protein kinase C gamma (PKCγ) is a signalling protein expressed in Purkinje cells (PCs) of the cerebellum. It is involved in regulating synapse formation and PC dendritic development through phosphorylation of target proteins that control cytoskeleton dynamics and PC dendritic morphology. Mutations in the protein kinase C gamma gene cause spinocerebellar ataxia 14 (SCA14), an autosomal dominant neurodegenerative disease leading to motor deficits and cognitive decline. The molecular targets of PKCγ and its interaction with cytoskeletal regulators are not yet well understood. Using a PKCγ mutant mouse line (PKCγ-A24E), which expresses a constitutively active PKCγ causing an SCA14-like phenotype, we identified Metastasis suppressor protein 1 (Mtss1) as a PKCγ molecular target involved in PC dendritic development. The two Mtss1 phospho-sites (S265 and S266) showed an increased phosphorylation in the cerebellum of PKCγ-A24E mice compared to wildtypes. Altering Mtss1-S265 and Mtss1-S266 with phospho-mimetic and phospho-defective mutations led to a loss of PC dendritic tree complexity in dissociated cerebellar cultures, with a developmental delay, a decreased dendritic branching, reduced spine density, and fewer synaptic processes, partially mimicking the PKCγ-A24E phenotype. Moreover, we found that these Mtss1 effects are partially regulated via the Arp2/3 complex. Our results demonstrate the importance of PKCγ-mediated phosphorylation of Mtss1 at S265 and S266 for PC dendritic outgrowth and suggest its contribution to PKCγ cytoskeleton signalling and the SCA14 phenotype.

**Supplementary Information:**

The online version contains supplementary material available at 10.1007/s12035-025-05526-9.

## Introduction

Purkinje cells (PCs) are the largest neurons of the cerebellar cortex presenting extensive dendritic trees that receive inputs from various cell types to refine the cerebellar output [1, 2]. The proper development of PC morphology is crucial for proper cerebellar function since disruptions in PC dendritic trees lead to impaired cerebellar circuits resulting in motor and cognitive disorders, including ataxias [3].

Spinocerebellar ataxia type 14 (SCA14) is a rare autosomal dominant neurodegenerative disease caused by mutations in protein kinase C gamma gene (PRKCG), encoding the PKCγ protein, leading to a progressive cerebellar atrophy and dysfunction characterized by motor impairments and cognitive decline [4]. To date, more than 75 mutations localized along the PRKCG gene have been reported to cause SCA14 [5, 6]. Depending on the mutation type (missense mutation, deletion, or nonsense mutation) and the location of the mutation within the PRKCG gene (pseudosubstrate domain, regulatory domain C1, calcium domain C2, and catalytic domain C3 or C4), some mutations trigger a gain of function of PKCγ [7] by impairing PKCγ autoinhibition [8], whereas other mutations may not increase PKCγ activity or even trigger a loss of function of PKCγ [9, 10]. These statements suggest that mutations in PRKCG gene may cause SCA14 by different mechanisms, and currently, no specific treatment exists to cure SCA14 [4] and other SCA subtypes [11].

PKCγ is a signalling protein kinase expressed predominantly in PCs of the cerebellum and involved in various neuronal processes, including the regulation of synapse formation and PC dendritic development [12]. The effects of PKCγ are mediated by phosphorylation of target proteins which regulate calcium signalling and cytoskeleton dynamics playing crucial roles in dendritic development [13, 14]. We have previously shown that PKCγ is a powerful mediator of dendritic development [14] and we successfully identified some PKCγ target molecules involved in PC dendritic development, such as CRMP2 [15], mGluR1 [16], and STK17b [17]. However, the specific PKCγ interactors with the neuronal cytoskeleton remained unclear. We here take advantage of a previously established PKCγ-A24E mouse line that contains a missense mutation in the pseudosubstrate domain of PKCγ leading to a constitutively active PKCγ and showing a perturbed PC maturation and ataxic behavior [18]. We used this SCA14 mouse model in order to identify PKCγ protein targets involved in PC development, spine formation, and synaptic initiation. Using phospho-proteomics, we identified Mtss1 as a molecular target of PKCγ, with two Mtss1 phospho-sites (S265 and S266) showing increased phosphorylation in the cerebellum of PKCγ-A24E mice compared to wildtype (WT).

Mtss1 is well-known to act as a tumor suppressor gene in different types of cancer [19, 20] since it controls cell proliferation, migration, invasion and metastasis of cancer cells [21] by regulating actin dynamics and signalling pathways [22, 23]. The role of Mtss1 in neural morphology arises from its interaction with cell membranes and the actin cytoskeleton through its actin-binding WH2 domain [24], which promotes dendritic filopodia protrusions during neurodevelopment [23, 25]. The indirect interaction of Mtss1 and the actin cytoskeleton has been studied also in Purkinje cells (PCs) of the cerebellum linking Mtss1 with Arp2/3 and formins to regulate dendritic filopodia and determine the final dendritic configuration [26]. Moreover, Mtss1 was identified as an ataxia gene linked to multiple spinocerebellar ataxias (SCAs) positioning it as a potential therapeutic target for SCAs [27, 28]. However, little is known about Mtss1 upstream regulators and molecular mechanisms involved in the ataxic phenotype.

Our findings demonstrate that PKCγ-mediated phosphorylation of Mtss1 at S265 and S266 is critical for maintaining the complexity of PC dendritic trees, spine density, and synaptic processes, being partially regulated through the Arp2/3 complex. Our results suggest that the Mtss1-Arp2/3 axis could serve as an intermediate link between PKCγ and the cytoskeleton, modulating PC development.

## Materials and Methods

### Animals

The knock-In mouse line PKCγ-A24E was previously described [18] and maintained in a FVB/NJ background. Both male and female mice were included in all experiments, and sex differences were not considered. All animals were kept under specified hygiene standards and according to animal welfare legislation. The experiments were executed in accordance with the EU Directive 2010/63/EU for animal experiments and approved by Swiss authorities.

### Cell Culture, Transfections, and Pharmacological Treatment

Human embryonic kidney cells (HEK293T) (RRID:CVCL_0063; ATCC; Molsheim Cedex, France) were grown under standard conditions (37˚C, 5% CO_2_). The growth medium DMEM (1X) + GlutaMAX™-I (61,965–026, Gibco) with 10% FBS (26,140,079, Gibco) and 1% Penicillin–Streptomycin (P4333-100ML, Sigma-Aldrich) was refreshed every 3–4 days. Cells were transfected at 60–80% confluency using the non-liposomal polymeric system TransIT-X2 Dynamic Delivery System (MIR 6000, Mirus) in reduced-serum Opti-MEM® I (1X) + GlutaMAX™-I medium (51,985–026, Gibco), following the manufacturer’s instructions. The plasmids used were: pCMV-PKCγ-WT-GFP, pCMV-PKCγ-A24E-GFP, pCMV-Mtss1-WT-myc, and pCMV-mock (empty vector). Transfected cells were maintained for 48 h. To modulate PKCγ activity, cells were treated with 300 nM of PMA (PKCγ activator, 1201, Tocris BioScience), 10 μM of Gö6983 (PKCγ inhibitor, 2285, Tocris BioScience), or 0,1% DMSO control, and incubated at 37˚C and 5% CO_2_ for 1–6 h before protein collection, depending on the experiment.

### Dissociated Cerebellar Cultures, Transfections, and Pharmacological Treatment

Dissociated cerebellar cultures (DCCs) were carried out as previously described [29, 30]. In short, postnatal day 0 (P0) FVB/NJ mice were sacrificed by decapitation. The cerebellar primordium of each pup was extracted, placed in ice cold Hank’s Balanced Salt Solution (HBSS) and cut into approximately 1 mm^2^ pieces. The cerebellar tissues from approximately 3–5 pups were digested by adding 300 µl of freshly prepared 0.22μm filtered ice-cold papain solution (LK003176, Worthington) and incubated at 37 ˚C for 20 min. The digestion was stopped with 5% FBS in 500 µl of HBSS previously warmed to room temperature (RT) and the solution was centrifuged at 600 rpm for 4 min. The pellets were triturated with 350 µl of freshly prepared DNase I from bovine pancreas (D5025-150KU, Sigma-Aldrich) by pipetting carefully up and down. Three hundred fifty microliters of HBSS were added to the dissociated cells which were then harvested by centrifugation at 600 rpm for 4 min. The pellets were washed twice with 1 ml of HBSS and transferred into the transfection tubes with 10 µl of an L7-based plasmid (1µg/µl) and 90 µl of transfection Solution Set P3 for Primary cultures (PBP3-02250, Lonza Walkersville). Transfections were performed according to the manufacturer’s instructions, cells were resuspended with 300 µl of DMEM/F-12 (1:1) (1X) (21,331–020, Gibco) with 1X B-27 Supplement (17,504–044, Gibco), 1X GlutaMAX™-I (35,050–061, Gibco), 1X L-Glutamine 200mM (25,030–081, Gibco) and 10% FBS, and plated on Poly-D-Lysine/Laminin 12 mm #1 German Glass Coverslips (354,087, Corning® BioCoat®). After 2–3 h incubation at 37 ˚C and 5% CO_2_, 500 µl of serum-free medium was added. Cultures were maintained for 21 days in vitro*,* and medium was changed every 4–5 days with DMEM/F-12, 1X B-27 Supplement and 1X GlutaMAX™-I. For experiments inducing Arp2/3 complex inhibition, the DCCs were treated with 20 nM CK-666 (182,515, Merck Millipore) or 0,1% DMSO as a control. The pharmacological treatment began on day in vitro (DIV)14–15 and was continued until fixation at DIV21.

### Plasmid Generation

The plasmids containing PKCγ-WT (pCMV-PKCγ-WT-GFP) and PKCγ-A24E (pCMV-PKCγ-A24E-GFP for HEK293T cells and pL7- PKCγ-A24E-mGFP for PCs) constructs were previously described [18].

Mouse Metastasis suppressor 1 (Mtss1) transcript variant 1 (Origene, MC201978) in a tag-free pCMV-Kan/Neo (PCMV6KN) vector was amplified by PCR using CloneAmp HiFi PCR Premix (Takara, 639,298) and primers Mtss1 pL7 (NotI) forward: 5' TAT TGC GGC CGC ATG GAG GCT GTG ATC GAG AAG 3' and Mtss1 pL7 (NheI) reverse: 5’ TTA TGC TAG CTC AGA GAA GCG CGG TGC TG 3’. The pL7-mGFP vector [30] was linearized with NotI-HF (New England BioLabs, Ipswich, USA; R3189S) and NheI-HF (New England BioLabs, Ipswich, USA; R3131S) and ligated with the PCR product to make pL7-Mtss1-WT-mGFP. The ligation reaction was transformed into NEB Stable Competent Cells (New England Biolabs, C3040I) and plated onto 100 µg/ml ampicillin agar plates.

The resulting pL7-Mtss1-WT-mGFP plasmid served as a template to amplify Mtss1 using primers CMV Mtss1 IF F 5' TTCGTCGACTGGATCCATGGAGGCTGTGATCGAGAAGG 3' and CMV Mtss1 IF R 5' TCTGCTCGAGCGGCCGCTTAGAGAAGCGCGGTGCTGAGC 3' to create pCMV-Mtss1-WT-myc. The pCMV-myc vector was digested with BamHI-HF (New England BioLabs, Ipswich, USA; R3136S) and NotI-HF (New England BioLabs, Ipswich, USA; R3189S) and the PCR product was integrated into the linearized vector using In-Fusion® HD multiple-insert cloning protocol (Takara, 638,948). The In-Fusion reaction was transformed into Stellar Competent Cells (Takara, 636,763) and plated on LB agar containing 50 µg/ml kanamycin.

Q5® Site-Directed Mutagenesis Kit (E0554S, New England BioLabs) was used to insert S265D, S266D, S265D-S266D, S265A, S266A, and S265A-S266A mutations into Mtss1 construct with the following primers specified in Table 1 and using pL7-Mtss1-WT-mGFP as a template for the PCR. After the construct amplification with Q5 Hot Start High-Fidelity Master Mix following the instructions of the provider, the PCR products were incubated with KLD enzyme mix for 5 min at RT followed by a transformation into NEB Stable Competent Cells (New England Biolabs, C3040I) by Standard cloning procedures.

**Table 1:** Primers used for site-directed mutagenesis

Plasmid name	Forward primer 5’−3’	Reverse primer 5’−3’
pL7-Mtss1-S265D-mGFP	GACGCCTCCT**GA**TTCTCCCAGTACAAC	TGATAGGACCAACTATAG
pL7-Mtss1-S266D-mGFP	GACGCCTCCTTCT**GA**TCCCAGTACAAC	TGATAGGACCAACTATAG
pL7-Mtss1-S265D-S266D-mGFP	GACGCCTCCT**GA**T**GA**TCCCAGTACAAC	TGATAGGACCAACTATAG
pL7-Mtss1-S265A-mGFP	TGTCCAGAAAGTCAAGTGTCTGCAGC	TAGTTGTACTGGGAGAAG**C**AGGAGGCGTCT
pL7-Mtss1-S266A-mGFP	TGTCCAGAAAGTCAAGTGTCTGCAGC	TAGTTGTACTGGGAG**C**AGAAGGAGGCGTCT
pL7-Mtss1-S265A-S266A-mGFP	TGTCCAGAAAGTCAAGTGTCTGCAGC	TAGTTGTACTGGGAG**C**AG**C**AGGAGGCGTCT

All primer sequences are presented in the 5’ to 3’ orientation, with mutations indicated in bold.

### Protein Extraction of Cerebellar Samples

Briefly, P7, P14, P21, P35, and P266 FVB/NJ mice and P14 PKCγ-A24E^wt/wt^, PKCγ-A24E^wt/+^, and PKCγ-A24E^+/+^ mice (3 mice per condition) were sacrificed by decapitation, the cerebellum of each mouse was extracted, transferred into a 1,5 ml tube, frozen with liquid nitrogen, and stored at −80 ˚C until all samples of the same experiment were collected. The cerebellums were transferred into Lysing Matrix D Tubes (6,913,100, MP Biomedicals) and lysed with 200–500 µl of RIPA buffer depending on the size of the cerebellum (50 mM Tris–HCl, pH 7,4; 0,15 M NaCl, 0,25% deoxycholic acid/sodium deoxycholate, 1% NP-40, 1 mM EDTA), containing protease inhibitors (cOmplete, EDTA-free 11,873,580,001, Sigma Aldrich) and phosphatase inhibitors (PhosSTOP EASY pack, 04906837001, Roche). The tissues were triturated using the FastPrep-24 5G homogenizer (MP Biomedicals) at 6 m/s for 40s. Then, the samples were centrifuged at 15.000 rpm for 15 min at 4 ˚C, and the supernatants were transferred into new 1,5 ml tubes and stored at −80 ˚C until usage.

### Protein Extraction of HEK293T Cells

Previously transfected and/or PMA-treated HEK293T cells were washed twice with PBS, transferred into 1,5 ml tubes and lysed with 100 µl of RIPA buffer containing protease inhibitors and phosphatase inhibitors. Then, the samples were sonicated (Bioruptor, 10 cycles, 30 s on/off, Diagenode) and centrifuged at 14.000 rpm for 10 min at 4 ˚C. The supernatants were stored at −80 ˚C until usage.

### Western Blot

Briefly, the lysate concentrations were determined by using a BCA protein assay kit (Invitrogen) and 2X Laemmli buffer was added before loading the samples into 8% polyacrylamide gels. Gels were run at 30 mA for 60–75 min in running buffer (0,25 M Tris, 1,93 M glycine, 0,1% SDS) and the proteins were transferred onto nitrocellulose membranes (1,620,115, Bio-Rad Laboratories) in transfer buffer (0,25 M Tris, 1,93 M glycine, 20% methanol) at 360 mA for 1 h. After blotting, the membranes were blocked with 5% BSA in tris-buffered saline (TBS) for 1 h shaking at RT and incubated with primary antibodies shaking overnight (ON) at 4˚C. Rabbit anti-Mtss1 (1:1000, Novus biologicals, NBP2-24,716), mouse anti-β-actin (1:2000, Sigma Aldrich, A5441), rabbit anti-phospho-(Ser) PKC substrate (1:1000, Cell Signaling technology, 2261), mouse anti-Mtss1 (1:200, Santa Cruz biotechnology, sc-101204), mouse anti-myc (1:1000, Origene, TA150121), mouse anti-PKCγ (1:1000, Proteintech, 66,429–1-Ig) were used. Then, the membranes were washed with TBS-0,1% Triton X-100 (TBST) and incubated with appropriate secondary antibodies; IRDye 800CW donkey anti-mouse (1:10,000, LI-COR Biosciences, 926–32 212) and IRDye 680LT donkey anti-rabbit (1:10,000, LI-COR Biosciences, 926–68 023) for 2 h shaking at RT. After washing with TBST, the signal was detected using the Odyssey® Fc Imaging device and software (LI-COR Biosciences).

### Immunoprecipitation (IP)

Immunoprecipitation of Mtss1 was performed using the Pierce® Crosslink Immunoprecipitation Kit (Thermo scientific, 26,147) following the manufacturer’s instructions. Briefly, protein lysates were prepared from HEK293T cells co-transfected with pCMV-PKCγ-WT-GFP and pCMV-Mtss1-WT-myc using the IP lysis/wash buffer provided in the kit. Mouse anti-myc (10 μg; Origene, TA150121) and mouse IgG (10 μg; Cell Signaling, 5415S) antibodies were incubated with protein A/G-agarose beads for 1 h at RT, and crosslinked with disuccinimidyl suberate for 1 h at RT. Lysates were pre-cleared with Control Agarose Resin slurry for 1 h at 4 °C to reduce non-specific binding. A total of 2000 μg of protein in 500 μl of IP lysis/wash buffer containing protease and phosphatase inhibitors was incubated ON at 4 °C with the antibody-bead conjugates on a rotating platform. Bound proteins were eluted under acidic conditions, neutralized with 1 M Tris buffer, denatured in 2 × Laemmli buffer, and analysed by Western blot.

### Immunohistochemistry of Cerebellar Sections

In short, P7, P14, P21, and P35 FVB/NJ mice and P14 PKCγ-A24E^wt/wt^, PKCγ-A24E^wt/+^, and PKCγ-A24E^+/+^ mice were euthanized with Esconarkon (150 mg/kg) (Streuli Tiergesundheit SA) and perfused by PBS plus 50 units/ml Heparin followed by fixation with 4% PFA plus 5% sucrose. The brains were extracted and post-fixed ON in 4% PFA and 30% sucrose. Then, the brains were frozen after embedding in Tissue-Tek (Tissue-Tek® O.C.T.™ Compound, Sakura, Ca. No. SA62550-01) at −40 ˚C in isopentane and 20 µm sagittal cryostat sections were cut with a Leica CM1950 cryostat. The 20 µm free floating sections were immunostained with a blocking solution containing 3% NGS (Thermo Fischer, PCN5000) and 0,5% Triton X-100 in PBS with the appropriate primary antibodies. Rabbit anti-Mtss1 (1:1000, Novus biologicals, NBP2-24,716) and guinea pig anti-Calbindin D28K (1:1000, Synaptic Systems, 214,004) were added into the floating sections and incubated ON shaking at 4 ˚C. After three washes with PB, the sections were incubated for 2 h shaking at RT with the relevant secondary antibodies; goat anti-rabbit Alexa 488 (1:500, Invitrogen, A11008) and goat anti-guinea pig Alexa 568 (1:500, Invitrogen, A11075) diluted in 0,1% Triton X-100 in PB. The sections were washed three times with PB and mounted with Mowiol. Images were acquired with a confocal laser scanning microscope (Zeiss LSM700) and adjusted for brightness and contrast.

### Immunohistochemistry of DCCs

DCCs were fixed with 4% PFA for 1 h at 4˚C, washed three times with PB, and immunostained with a blocking solution containing 3% NGS and 0,5% Triton X-100 in PBS with the appropriate primary antibodies incubated for 1 h at RT. After 3 washes with 0,1% Triton X-100 in PB, secondary antibodies were incubated for 1 h at RT, followed by 3 more washes with 0,1% Triton X-100 and the coverslips were mounted with Mowiol.

Rabbit anti-Calbindin D28K (1:1000, Swant, CB38a) and mouse anti-GFP IgG2a (1:1000, Acris, R1461P) were combined with goat anti-rabbit Alexa 568 (1:500, Invitrogen, A11011) and goat anti-mouse Alexa 488 (1:500, Invitrogen, A11001), respectively. Guinea pig anti-Calbindin D28K (1:1000, Synaptic Systems, 214,004), rabbit anti-Mtss1 (1:1000, Novus biologicals, NBP2-24,716), and mouse anti-Arp2(E-12) (1:500, Santa Cruz Biotechnology, sc-166103) were combined with goat anti-guinea pig Alexa 350 (1:500, Sigma Aldrich, SAB4600012), goat anti-rabbit Alexa 568 (1:500, Invitrogen, A11011), and goat anti-mouse Alexa 488 (1:500, Invitrogen, A11001), respectively. Goat anti-GFP (1:1000, abcam, ab6673), rabbit anti-VGLUT1 (1:1000, Synaptic Systems, 135,302), and mouse anti-PSD-95 (7E3-1B8)(1:200, Invitrogen, MA1-046) were combined with donkey anti-goat Alexa 488 (1:500, Invitrogen, A32814), goat anti-rabbit Cy5 (1:500, Jackson ImmunoResearch, 111–175-144), and goat anti-mouse Alexa 568 (1:500, Invitrogen, A11004), respectively.

### Sholl Analysis

Images were acquired with an Olympus AX-70 microscope with a 20X objective. For the analysis of dendritic arbours, the images of GFP-labelled PCs were smoothed and Sholl analysis was performed using the Sholl analysis plugin in Fiji ImageJ software [31]. The starting radius of the Sholl analysis was 15 µm and the shells surrounding the cell soma had 5 µm distance from each other. From the Sholl analysis we plotted the number of intersections from the soma to the end of the cell and we summed the total number of intersections per cell. The area covering the dendritic tree was measured with the area limited to threshold and the last shell detecting intersections was measured as the longest dendrite of the cell. The number of primary dendrites was manually counted. The identity of all analysed PCs was confirmed by Calbindin staining.

### Spine Analysis

Images were acquired with an inverted point-scanning confocal Nikon AxR microscope with NSPARC array detector and 100X oil immersion objective. For each experiment, images were acquired using the same detection filter settings, pinhole, and pixel size, with a z-interval of 0,17 µm. The maximum projection of z-stack images for each GFP-labelled PC was analysed using the Dendritic spine counter plugin in Fiji ImageJ software. Analysis was performed only from distal dendrites (around 8–10 dendrites/cell) and following the instructions of the plugin. The neck length, the neck width, and the head width were measured by hand by drawing lines at each spine. Spine density was normalized by the length of the dendrite. The spine length and head width were averaged by dendrite. The classification of spines was performed with an algorithm previously described [32] and normalized by the total number of spines multiplied by 100. Briefly, dendritic spines were categorized manually measuring the length (L), head width (H), and neck width (N) of individual spines. Spines were classified quantitatively into filopodia (H/N < 1.2 and L/N > 3), mushroom (H/N ≥ 1.5), stubby (H/N ≤ 1 and L/N ≤ 1), or thin (H/N 1 ≤ ≥ 1.5 and L/N 1.5 ≤ ≥ 3) types. Spines that did not meet any of these criteria were classified as unclassified and still included in the analysis. The identity of all analysed PCs was confirmed by Calbindin staining.

### Quantification of Synaptic Processes

Images were acquired with an inverted point-scanning confocal Nikon AxR microscope with NSPARC array detector and 100X oil immersion objective. For each experiment, images were acquired using the same detection filter settings, pinhole, and pixel size, with a z-interval of 0,17 µm. The maximum projection of z-stack images for each GFP-labelled PC was used for the analysis. The quantification of synaptic processes was analysed with a previously designed Script in Fiji ImageJ software [33]. In short, the number of active synapses was measured by counting the number of processes in which the VGLUT1 signal was overlapping with the PSD-95 signal followed by a normalization by the area of the dendrite. To identify active synaptic processes, the signal of the markers was filtered based on intensity (≥ 50% overlap between markers) and size (objects ≤ 3 μm^2^). To determine the percentage of synaptic area, the PSD-95-VGLUT1 overlapping area was divided by the dendritic area of the studied dendritic segment and multiplied by 100. The identity of all analysed PCs was confirmed by Calbindin staining.

### Proteomics and Phospho-Proteomics

Briefly, P21 PKCγ-A24E^wt/wt^, PKCγ-A24E^wt/+^, and PKCγ-A24E^+/+^ mice were sacrificed by decapitation and cerebella were isolated and quickly frozen in liquid nitrogen. Cerebellar proteins were extracted with lysis buffer containing 8M urea, 0.1 M ammonium bicarbonate, protease inhibitors (cOmplete, EDTA-free 11,873,580,001) and phosphatase inhibitors (PhosSTOP EASY pack, 04906837001, Roche). Protein concentrations were determined by a BCA protein assay kit (Invitrogen).

For proteomics, sample aliquots comprising 12.5 μg of peptides were labelled with isobaric tandem mass tags (TMT 10-plex, Thermo Fisher Scientific), resuspended in 0.1% aqueous formic acid, and subjected to LC–MS/MS analysis. For phospho-proteomics, proteins were digested and peptide samples were enriched for phosphorylated peptides using Fe(III)-IMAC cartridges as previously described [18]. Phospho-enriched peptide samples were also resuspended in 0.1% aqueous formic acid and subjected to LC–MS/MS analysis. LC–MS was performed by Alexander Schmidt using an Orbitrap Fusion Lumos Mass Spectrometer fitted with an EASY-nLC 1200 (Thermo Fisher Scientific) in the Biocenter of the University of Basel, Switzerland. The analyses were normalized to total peptide and significantly altered proteins were identified by filtering for a protein upregulation or a phosphorylation increase in PKCγ-A24E^+/+^ compared to PKCγ-A24E^wt/wt^ with a p-value < 0,05.

### IPA Pathway Analysis

Significantly altered proteins in PKCγ-A24E^+/+^ mice compared to PKCγ-A24E^wt/wt^ based on proteomics, and proteins with significantly altered phosphorylation in PKCγ-A24E^+/+^ mice compared to PKCγ-A24E^wt/wt^ according to phospho-proteomics, were submitted to pathway analysis using the QIAGEN’s IPA software. The results of the core analysis showed direct and indirect relationships between altered proteins pointing the altered proteins at each altered pathway in PKCγ-A24E^+/+^ mice compared to PKCγ-A24E^wt/wt^.

### Statistical Analyses

Statistical analyses were performed using GraphPad Prism10 software. Normality was assessed using the Shapiro–Wilk test for small sample sizes (n < 50) and the Kolmogorov–Smirnov test for larger ones (n > 50). Variance was evaluated using the F-test, and unpaired T-tests were used for statistical comparison between two populations. Bartlett’s test was applied to assess differences in standard deviation (SD). For comparisons among more than two groups: if data were normally distributed without significant differences in SD, one-way ANOVA with Sidak’s multiple comparisons test was used; if data were not normally distributed, the Kruskal–Wallis test with Dunn’s multiple comparisons test was applied; and if SDs differed significantly, Brown-Forsythe and Welch ANOVA test with Dunnett’s T3 multiple comparisons test was used.

## Results

### Mtss1 Expression is Regulated by PKCγ Activity

**Fig. 1:**
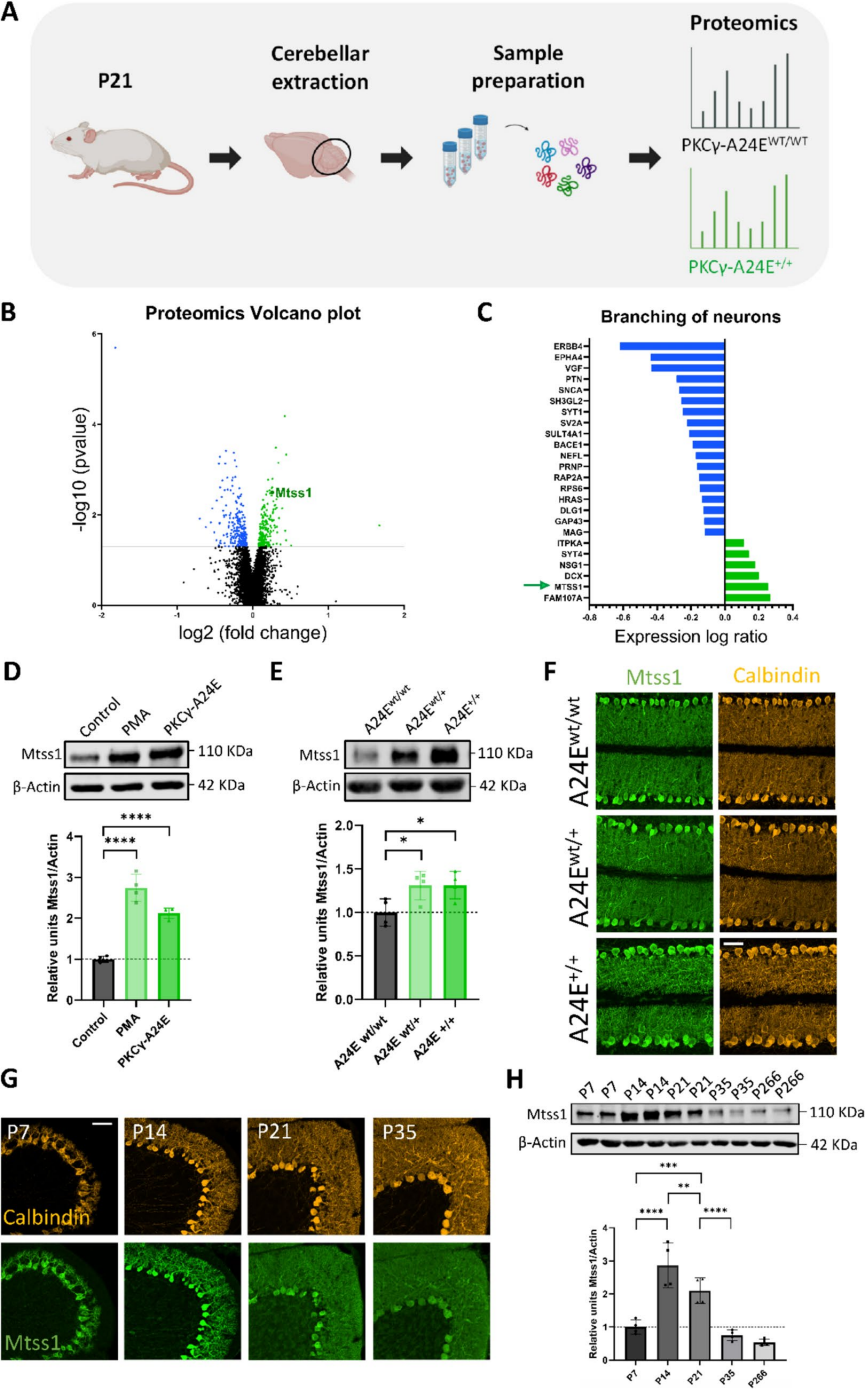
Mtss1 expression is regulated by PKCγ activity. (**A**) Proteomic analysis of cerebellar protein samples from P21 PKCγ-A24E^+/+^ and PKCγ-A24E^wt/wt^ mice was performed to profile protein differences between genotypes. (**B**) Volcano plot of proteomics analysis of PKCγ-A24E^+/+^ versus PKCγ-A24E^wt/wt^ mice (n = 3) at P21. Differentially enriched proteins are shown in the volcano plot; downregulated (left, blue) and upregulated (right, green). Mtss1 is among the proteins with a significant upregulation, pointed in green to differentiate it. X axis is log2 fold change and Y axis is p-value. (**C**) IPA showed that many differentially enriched proteins are involved in branching of neurons, including Mtss1 (pointed with a green arrow). Blue proteins represent downregulation and green proteins represent upregulation. (**D**) Western blot staining of Mtss1 and corresponding graph of Mtss1 relative expression normalized to Actin show Mtss1 protein upregulation in HEK293T cells co-transfected with PKCγ-WT and Mtss1-WT and treated with PMA for 6 h, and in HEK293T cells co-transfected with PKCγ-A24E construct and Mtss1-WT (Control = 1 ± 0,07; PMA = 2,74 ± 0,33; A24E = 2,12 ± 0,13). (**E**) Western blot analysis of whole cerebellar lysates shows Mtss1 protein upregulation in PKCγ-A24E^+/+^ (hom) and PKCγ-A24E^wt/+^ (het) mice compared to PKCγ-A24E^wt/wt^ (wt). Relative protein quantification of Mtss1 was normalized to Actin. P14, n = 4: wt = 1 ± 0,16; het = 1,30 ± 0,17; hom = 1,31 ± 0,16. (**F**) Representative images of sagittal cerebellar sections of lobules V-VI comparing PKCγ-A24E^wt/wt^, in PKCγ-A24E^wt/+^, and in PKCγ-A24E^+/+^ at P14. Protein distribution of Mtss1 and Calbindin displayed in green and yellow, respectively. (**G**) Representative images of WT sagittal cerebellar sections of lobule VIb at P7, P14, P21, and P35. Protein distribution of Mtss1 (green) and Calbindin (yellow) is shown. (**H**) Western blot analysis of WT cerebellar lysates over time shows the highest Mtss1 expression at P14. Mtss1 protein quantification was normalized to Actin. P7 = 1 ± 0,21; P14 = 2,87 ± 0,68; P21 = 2,09 ± 0,39; P35 = 0,75 ± 0,15; P266 = 0,54 ± 0,10; n = 4. Scale bar = 50 μm. Ordinary one-way ANOVA with post-hoc Sidak comparisons test; * = p-value < 0,05; ** = p-value < 0,01; *** = P-value < 0,001; **** = p-value < 0,0001. Error bars indicate SD

We profiled protein differences in PKCγ-A24E mutant mice compared to WTs by using proteomics of cerebellar protein samples of three-week-old pups (Fig. 1A). The Volcano plot data show upregulated (right side) and downregulated (left side) proteins in PKCγ-A24E homozygous mice compared to WTs, highlighting Mtss1 as a potential PKCγ target molecule significantly upregulated in PKCγ-A24E^+/+^ (homozygous) mice compared to WTs (Fig. 1B). Mtss1 is predicted to be involved in the branching of neurons (Fig. 1C), as well as in the overall morphogenesis of neurons and the morphology of the actin cytoskeleton (Fig. S1A-B) according to IPA pathway analysis. The increase of Mtss1 protein levels upon PKCγ overactivation was validated in HEK239T cells. Cells co-transfected with PKCγ-WT and Mtss1-WT and treated with PMA (PKCγ activator) showed a 2,72-fold increase (p-value = 0,0094), while cells co-transfected with PKCγ-A24E mutant construct and Mtss1-WT exhibited a 2,16-fold increase (p-value = 0,0286) compared to untreated PKCγ-WT/Mtss1-WT transfected cells (Control), as determined by Western blots with relative protein quantification (Fig. 1D). In the PKCγ-A24E mutant mice, we could validate the Mtss1 upregulation in PKCγ-A24E^wt/+^ (heterozygous) mice (1,30-fold increase, p-value = 0,0452) and PKCγ-A24E^+/+^ mice (1,31-fold increase, p-value = 0,0431) compared to PKCγ-A24E^wt/wt^, also determined by Western blots (Fig. 1E). Immunostainings confirmed that Mtss1 is highly expressed in PCs, following a similar pattern as calbindin (PC specific marker), although no major changes in protein localization were shown in the immunohistochemistry of cerebellar sections comparing PKCγ-A24E^wt/wt^, PKCγ-A24E^wt/+^, and PKCγ-A24E^+/+^ (Fig. 1F). In order to characterize Mtss1 in PCs of the cerebellum, we studied Mtss1 protein levels during different developmental stages until adulthood by Western blots and Immunostaining. Mtss1 was highly expressed in PCs during PC development reaching its highest expression at postnatal day 14 (P14) (2,87-fold increased expression compared to P7, p-value < 0,0001) followed by a progressive decline (protein relative values: P7 = 1; P14 = 2,87; P21 = 2,09; P35 = 0,75; P266 = 0,54) (Fig. 1G-H).

### Mtss1 Phosphorylation is Regulated by PKCγ Activity

**Fig. 2:**
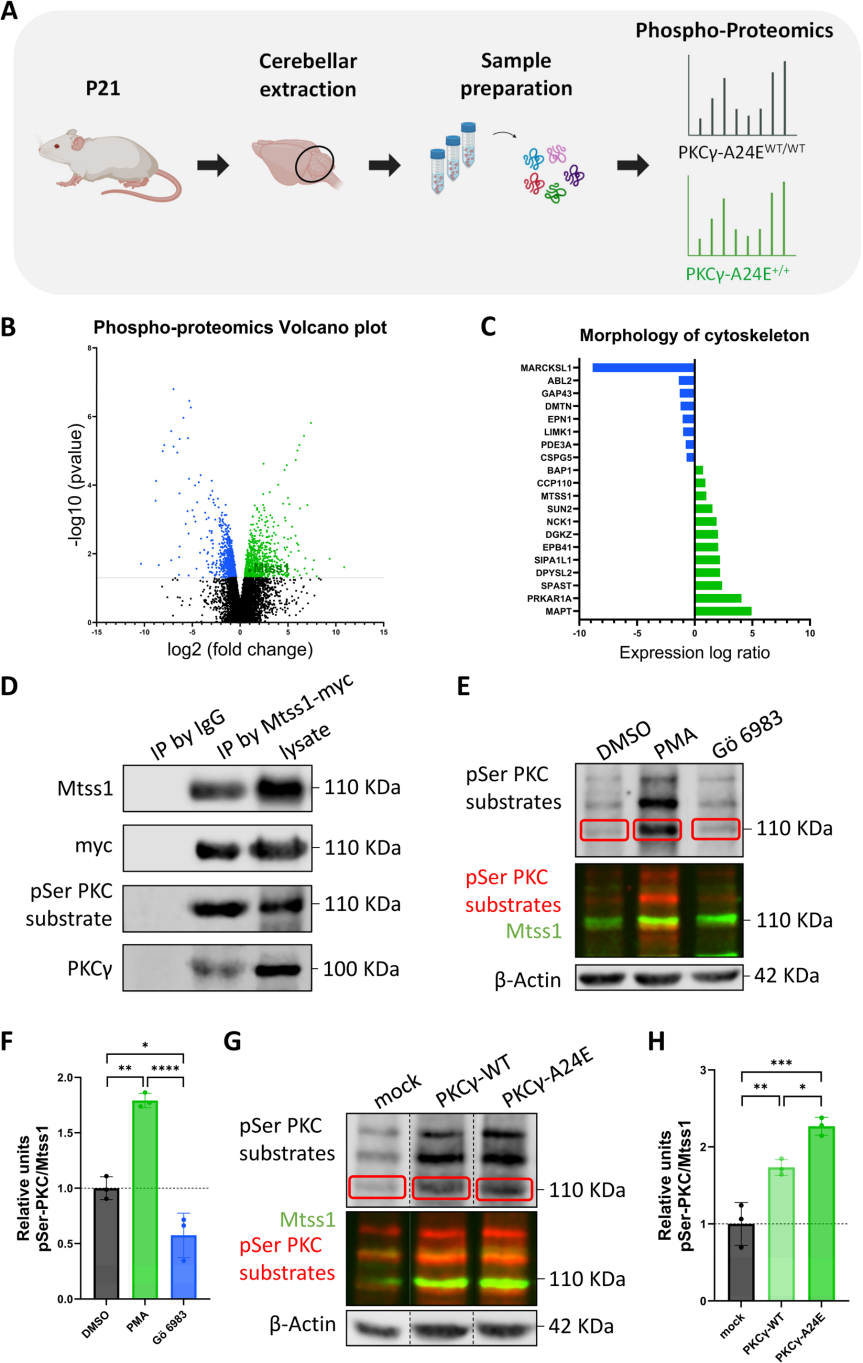
Phosphorylation of Mtss1 is increased in models with PKCγ overactivation. (**A**) To assess phosphorylation differences between PKCγ-A24E^+/+^ mutant and PKCγ-A24E^wt/wt^ mice, phospho-proteomic analysis was performed on cerebellar protein samples from P21 pups. (**B**) Volcano plot of phospho-proteomics analysis of PKCγ-A24E^+/+^ versus PKCγ-A24E^wt/wt^ mice (n = 3) at P21. Peptides differentially phosphorylated are shown in the volcano plot; decreased phosphorylation (left, blue) and increased phosphorylation (right, green). Mtss1 is among the proteins with a significant increased phosphorylation, pointed in dark green to differentiate it. X axis is log2 fold change and Y axis is p-value. (**C**) IPA showed that many proteins with an altered phosphorylation are involved in the morphology of the cytoskeleton, including Mtss1 (pointed with a green arrow). Blue proteins represent a decrease in phosphorylation whereas green proteins represent an increase. (**D**) Immunoprecipitation demonstrates that Mtss1 is associated with PKCγ and is detected as a phospho-serine (pSer) PKC substrate. (**E**) Immunoblot of pSer PKC-substrates overlapping with Mtss1 expression in lysates of HEK293T cells co-transfected with PKCγ-WT and Mtss1-WT, and treated for 1 h with 300 nM PMA, 10 μM Gö6983, or 0,1% DMSO (Control). (**F**) Corresponding quantification showing a significant increase or decrease in pSer-Mtss1 relative protein expression, normalized to Actin and to Mtss1, in cells treated with PMA or Gö6983 compared to DMSO controls, respectively (DMSO = 1 ± 0,10; PMA = 1,79 ± 0,07; Gö6983 = 0,57 ± 0,20). (**G**) Western blot of HEK293T cells co-transfected with Mtss1-WT and either mock (empty vector), PKCγ-WT, or PKCγ-A24E constructs. (**H**) Corresponding quantification showing a significant increase in pSer-Mtss1 relative expression, normalized to Actin and to Mtss1, in cells co-transfected with Mtss1-WT and PKCγ-A24E compared to controls (mock and PKCγ-WT) (mock = 1 ± 0,28; PKCγ-WT = 1,73 ± 0,10; PKCγ-A24E = 2,27 ± 0,12). Red squares in (**E**) and (**G**) indicate the pSer PKC substrate signal corresponding to Mtss1 (~ 110 KDa). One-way ANOVA; * = p-value < 0,05; ** = p-value < 0,01; *** = P-value < 0,001; **** = P-value < 0,0001. Error bars indicate SD

The protein phosphorylation profile of PKCγ-A24E mice compared to WTs using phospho-proteomics with cerebellar protein samples of seven-week-old mice was previously described [18]. With the aim to better understand the PKCγ molecular pathway during PC development, we now used phospho-proteomics for comparing three-week-old PKCγ-A24E mice vs WT mice (Fig. 2A). The Volcano plot shows the increased phosphorylation of two Mtss1 phospho-sites (S265 and S266) in PKCγ-A24E^+/+^ mice compared to PKCγ-A24E^wt/wt^ (Fig. 2B). IPA pathway analysis from phospho-proteomics data predicted that many proteins with an altered phosphorylation are involved in the morphology of the cytoskeleton (Fig. 2C), branching of neurites (Fig. S2A), and formation of filopodia (Fig. S2B), emphasizing the critical role of protein phosphorylation in regulating neural morphogenesis. Mtss1 is one of the proteins with altered phosphorylation implicated in all three processes.

In the absence of phospho-specific antibodies for Mtss1, we immunoprecipitated Mtss1 from HEK293T cells co-transfected with pCMV-PKCγ-WT-GFP and pCMV-Mtss1-WT-myc, using an anti-myc antibody. With the purified Mtss1 IP sample, we could validate that Mtss1 is a phospho-serine PKC substrate, detected with the pSer PKC substrate antibody. Additionally, we observed a slight but relevant co-immunoprecipitation of PKCγ with Mtss1, suggesting a direct interaction between both proteins (Fig. 2D). The hyperphosphorylation of Mtss1 in cells with increased PKCγ activity was validated by detecting the increase of phospho-serine PKC substrates at the level of Mtss1 protein size, normalized by the total Mtss1 protein. This was assessed via Western blots of protein samples from HEK293T cells co-transfected with PKCγ-WT and Mtss1-WT, and treated with PMA, Gö6983, or DMSO as a control (Fig. 2E, F). A substantial increase in phosphorylation in PMA-treated cells (1,79-fold increase, p-value = 0,0011) and a decrease in phosphorylation in Gö6983-treated cells (0,57-fold increase, p-value = 0,0251) was observed. In parallel, co-transfection of HEK293T cells with Mtss1-WT and either mock, PKCγ-WT, or PKCγ-A24E constructs confirmed a marked increase in phosphorylation in PKCγ-A24E transfected cells compared to both PKCγ-WT (1,30-fold increase, p-value = 0,036), and mock (1,73-fold increase, p-value = 0,0005) (Fig. 2G, H).

### Purkinje Cell (PC) Dendritic Morphology is Regulated by Mtss1 Phosphorylation

**Fig. 3:**
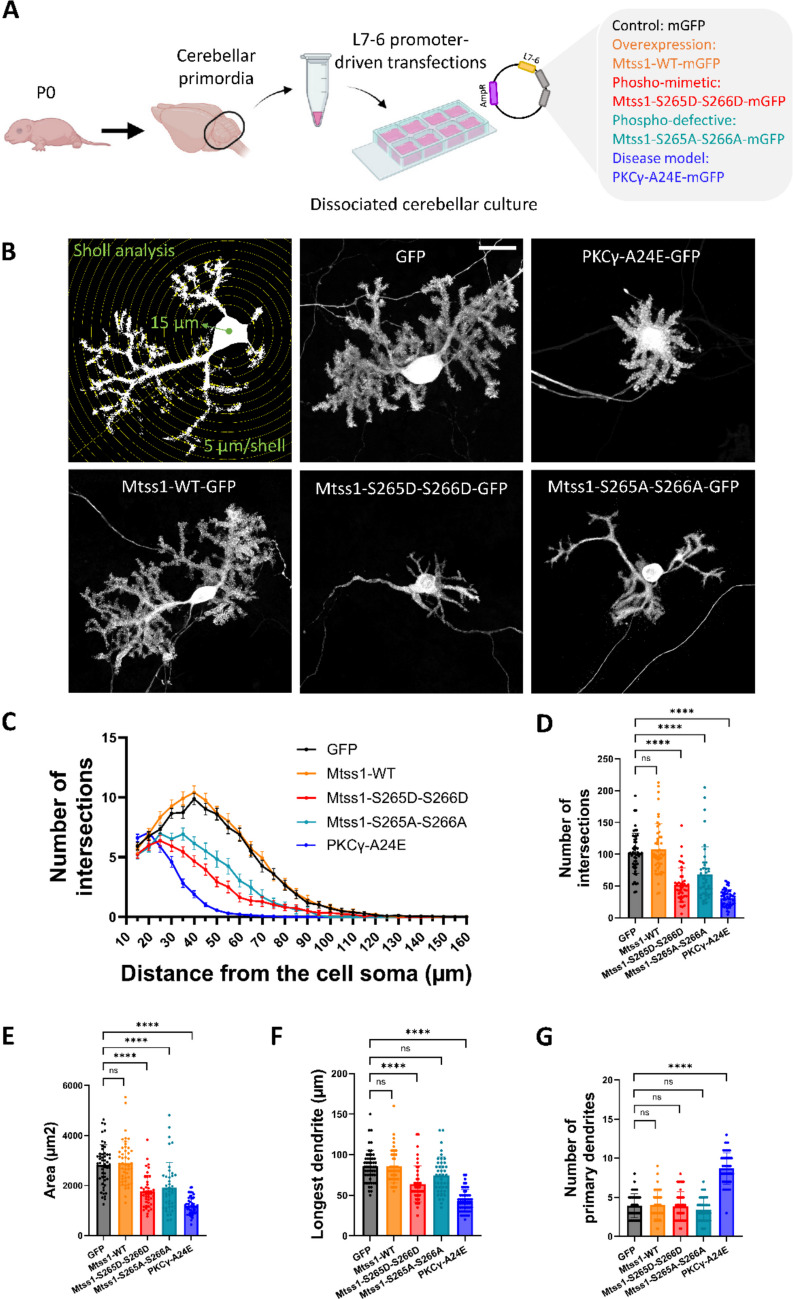
PC dendritic morphology is regulated by Mtss1 phosphorylation. (**A**) Overview of dissociated cerebellar cultures transfected with L7-6 promoter-driven plasmids to assess the effects of Mtss1 phosphorylation on PC dendritic development. (**B**) Schematic of Sholl analysis showing the first shell surrounding the cell soma in a radius of 15 μm followed by 5 μm distance with each next shell (top left). Representative images of GFP-control, PKCγ-A24E-GFP, Mtss1-WT-GFP, Mtss1-S265D-S266D-GFP, and Mtss1-S265A-S266A-GFP transfected PCs in DCCs at DIV21. Fluorescence detection: GFP. Scale bar = 20 μm. (**C**) Sholl analysis showing the number of intersections from the soma to the end of the cell (data represented as mean ± SEM), (**D**) total number of intersections, (**E**) area, (**F**) longest dendrite, and (**G**) number of primary dendrites of PCs transfected with GFP (n = 52 cells), Mtss1-WT (n = 48 cells), Mtss1-S265D-S266D (n = 43 cells), Mtss1-S265A-S266A (n = 42 cells), and PKCγ-A24E (n = 51 cells). Brown-Forsythe and Welch ANOVA test with post-hoc Dunnett comparisons test and Kruskal–Wallis test with post-hoc Dunn comparisons test; * = p-value < 0,05; ** = p-value < 0,01; *** = P-value < 0,001; **** = p-value < 0,0001. Error bars (**D-G**) indicate SD. Data produced in 3–5 independent cultures per condition

In order to study the role of Mtss1 phosphorylation in PCs, we studied PCs in DCCs after transfection with Mtss1-constructs affecting Mtss1 phosphorylation in different ways (Fig. 3A). The overexpression of Mtss1-WT-mGFP fusion protein did not yield any change in PC dendritic arbours compared to control GFP transfected PCs. However, PCs transfected with Mtss1 containing phospho-mimetic (Mtss1-S265D-S266D) or phospho-defective (Mtss1-S265A-S266A) mutations triggered the loss of PC dendrite complexity, partially mimicking the SCA14 phenotype in vitro as shown in PCs transfected with PKCγ-A24E constructs (Fig. 3B). Sholl analysis was performed in order to confirm these morphological changes (Fig. 3C). Mtss1-S265D-S266D and Mtss1-S265A-S266A PCs yielded significantly reduced number of intersections, with decreased area and reduced longest dendrite compared to GFP transfected PCs and Mtss1 transfected PCs (Fig. 3D-F). The alteration of both phospho-sites (S265-S266) at the same time is required to trigger the disruption of PC dendritic trees, since no significant changes were shown in the total number of intersections, the area, the longest dendrite, and the number of primary dendrites of PCs transfected with Mtss1 phospho-mimetic (S265D, S266D) and phospho-defective (S265A, S266A) single mutations compared to GFP controls (Fig. S3).

The loss of dendritic complexity was stronger in PCs transfected with PKCγ-A24E in terms of the number of intersections, area, and longest dendrite (Fig. 3D-F). Interestingly, although there were no significant changes in the number of primary dendrites in Mtss1-WT, Mtss1-S265D-S266D and Mtss1-S265A-S266A PCs compared to GFP transfected PCs, PKCγ-A24E transfected PCs yielded a significant increase in the number of primary dendrites (Fig. 3G). This increase in primary dendrites is unique for PKCγ-A24E suggesting that PKCγ-A24E disrupts PC dendritic development at an earlier developmental stage compared to Mtss1-S265D-S266D and Mtss1-S265A-S266A PCs.

### PKCγ-A24E Inhibits PC Dendritic Development at an Immature Stage, While Mtss1 Phosphorylation Reduces the Extension of Dendritic Branches

**Fig. 4:**
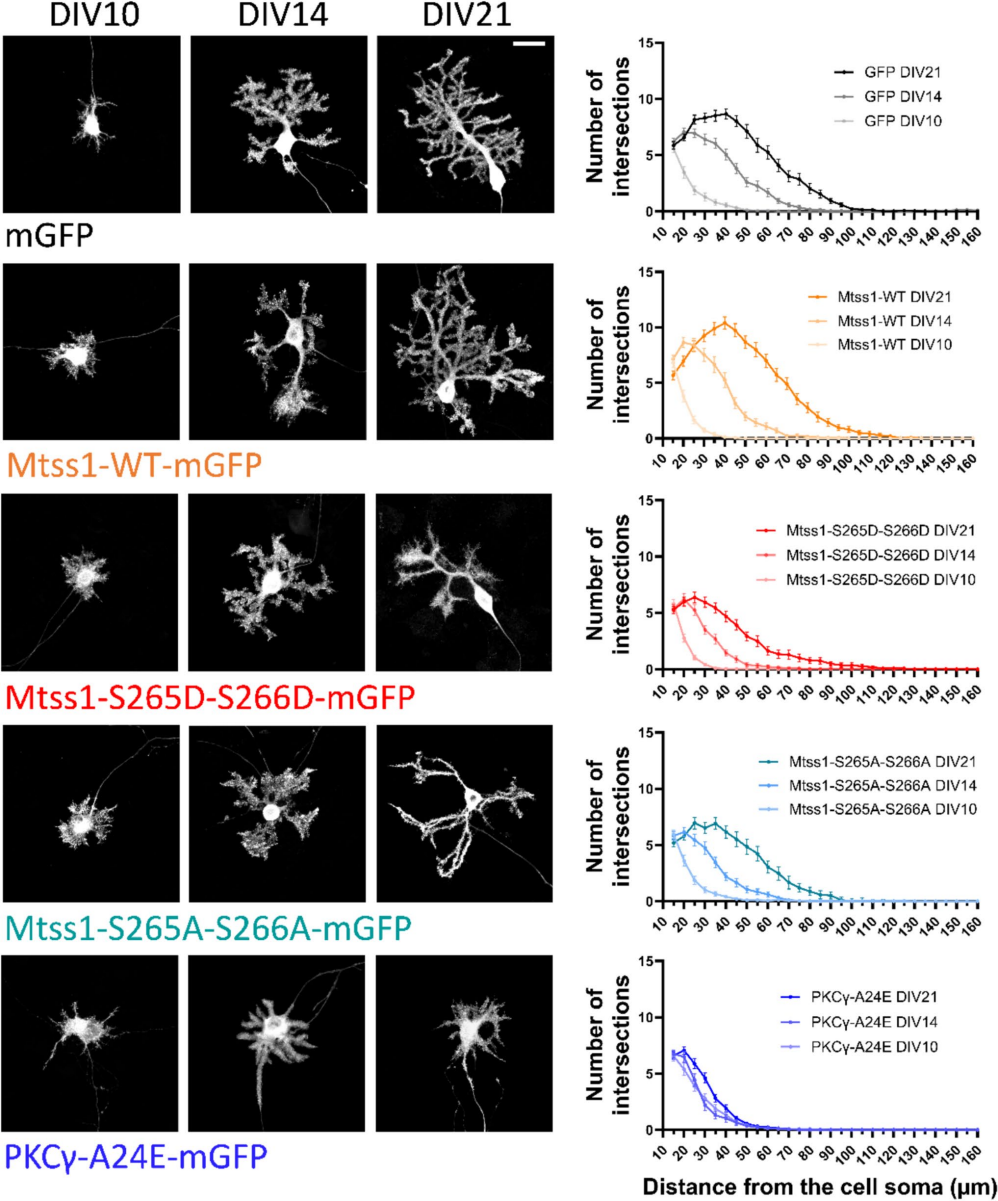
Mtss1 phosphorylation leads to a delay in PC dendritic development with an overall decreased dendritic outgrowth. Representative images of PCs at DIV10, DIV14, and DIV21, and the corresponding Sholl analysis comparing these 3 time points in PCs transfected with GFP, Mtss1-WT-GFP, Mtss1-S265D-S266D-GFP, Mtss1-S265A-S266A, and PKCγ-A24E. Fluorescence detection: GFP. Scale bar = 20 μm. GFP DIV10 n = 50 cells, DIV14 n = 47 cells, DIV21 n = 52 cells; Mtss1-WT DIV10 n = 52 cells, DIV14 n = 50 cells, DIV21 n = 48 cells; Mtss1 S265D-S266D DIV10 n = 57 cells, DIV14 n = 46 cells, DIV21 n = 43 cells; Mtss1-S265A-S266A DIV10 n = 51 cells, DIV14 n = 44 cells, DIV21 n = 42 cells; PKCγ-A24E DIV10 n = 45 cells, DIV14 n = 44 cells, DIV21 n = 51 cells. Data represented as mean ± SEM and produced in 3–5 independent cultures per condition

In order to better understand how PC dendritic arbours change with Mtss1 phospho-mutants, we studied PC morphology over time in vitro. Control-GFP PCs showed a stellate stage of development at day in vitro (DIV)10, in which the cell consists of the soma of the PC and several primary dendrites coming directly from the soma (also called neurites). At that stage there are no clear differences between the genotypes studied, apart from a slight increase in the number of intersections and in the length of the longest dendrite of PKCγ-A24E PCs compared to all the other genotypes (Fig. 4, S4 A-E, and S6 A-E). At DIV14, control-GFP PCs exhibit the retraction of most of the primary dendrites and the starting of dendritic branch development, until PCs are fully developed at DIV21. Mtss1 transfected PCs show a similar pattern as GFP-controls at DIV14 and DIV21. PCs from Mtss1-S265D-S266D and Mtss1-S265A-S266A also show the retraction of primary dendrites and initiation of dendritic branch development, but the branches produced remain shorter and less developed compared to WT (Fig. 4, S5 A-E, and S6 A-E). At DIV21, this difference in branch extension has increased and the PCs with Mtss1 mutations now have clearly reduced branches, while the overall branching pattern is similar to WT. In contrast, PKCγ-A24E mutant PCs seemed to be fixed in the immature state observed at DIV10. There was only little further development of the dendritic trees of PKCγ-A24E PCs from DIV10 to DIV14 and DIV21, suggesting a developmental block at the stellate stage of development (Fig. 4 and S6 A-E). These results suggest that although Mtss1 phosphorylation partially mimics the SCA14 phenotype shown in PKCγ-A24E PCs in vitro producing a dendritic tree of reduced size and complexity, additional processes might be affected in the PKCγ-A24E PCs triggering a stronger phenotype and preventing PC development beyond rather immature stages.

### PC Dendritic Spine Morphology and Synapse Formation are Regulated by Mtss1 Phosphorylation

**Fig. 5:**
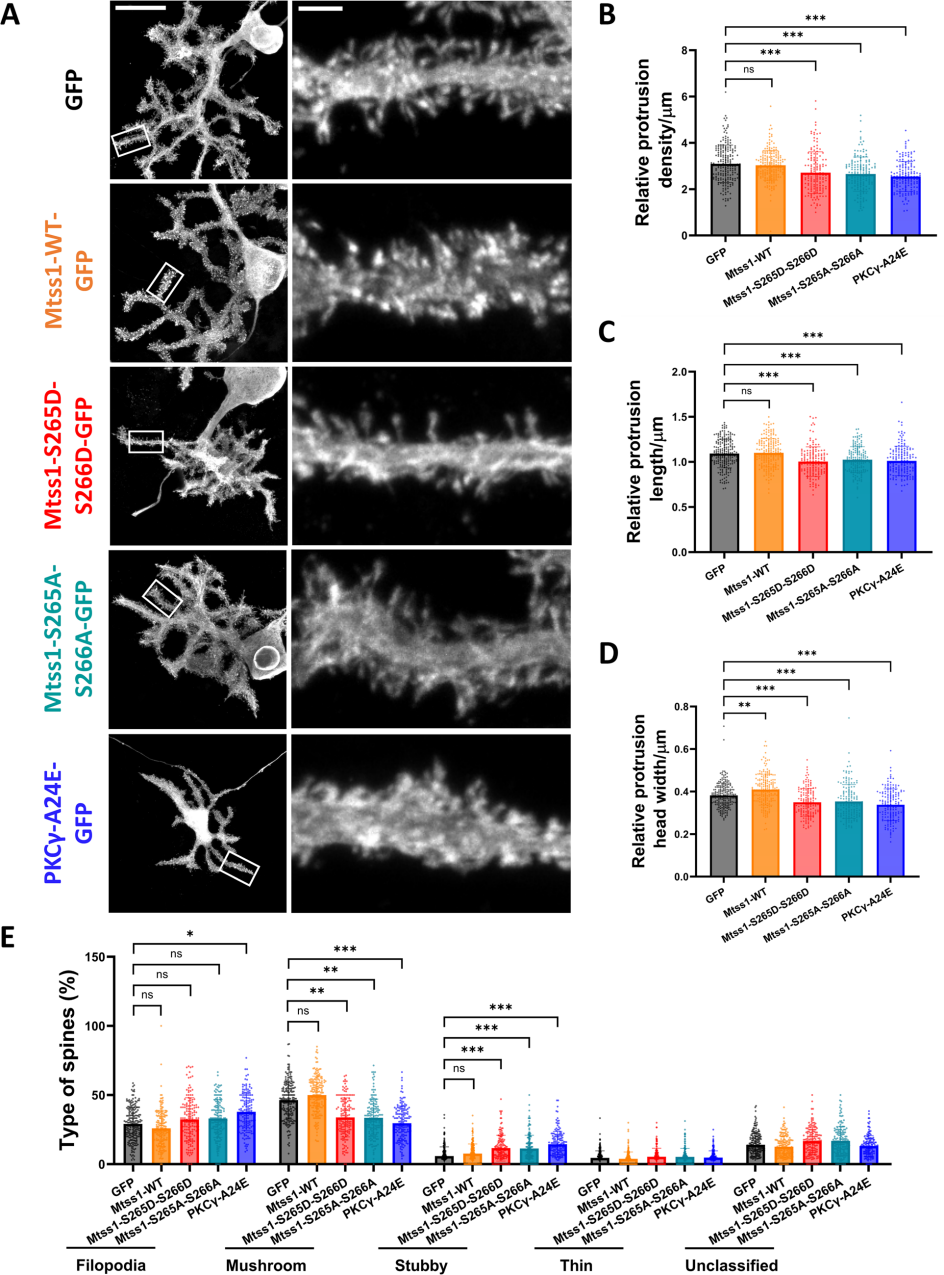
PC dendritic spine morphology is regulated by Mtss1 phosphorylation. (**A**) Representative images of GFP-labelled PCs at DIV21 (first column) comparing GFP, Mtss1-WT-GFP, Mtss1-S265D-S266D-GFP, Mtss1-S265A-S266A, and PKCγ-A24E, and higher-magnification dendritic segments (second column) to visualize spine morphology. Localization of selected dendritic segments pointed with a white box. Fluorescence detection: GFP. Scale bar = 20 μm/2 μm. (**B**) Protrusion density, (**C**) protrusion length, (**D**) protrusion head width, and (**E**) percentage of types of spines classified as filopodia, mushroom, stubby, thin, or unclassified spines comparing GFP, Mtss1-WT-GFP, Mtss1-S265D-S266D-GFP, Mtss1-S265A-S266A, and PKCγ-A24E. Brown-Forsythe and Welch ANOVA test with post-hoc Dunnett comparisons test and Kruskal–Wallis test with post-hoc Dunn comparisons test; * = p-value < 0,05; ** = p-value < 0,01; *** = P-value < 0,001; **** = p-value < 0,0001. Error bars indicate SD. GFP N = 26 cells, n = 204 dendrites; Mtss1-WT N = 24 cells, n = 191 dendrites; Mtss1-S265D-S266D N = 20 cells, n = 151 dendrites; Mtss1-S265A-S266A N = 20 cells, n = 156 dendrites; PKCγ-A24E N = 20 cells, n = 154 dendrites. Data results from 5–8 dendritic segments/cell; 4–8 cells/culture; 3–6 independent cultures per condition

Mtss1 was previously linked to dendritic protrusions since a PC specific Mtss1-KO triggered an increase in protrusion length whereas the Mtss1 overexpression in PCs in vitro triggered the opposite effect [26]. Therefore, we wanted to further study the effect of Mtss1 phosphorylation at the dendritic spine level in vitro (Fig. 5A-D). In our experiments, the spines of PCs with Mtss1-WT overexpression exhibited similar characteristics to those of Control-GFP PCs, with no significant differences in protrusion density, length, or the percentage of different types of spines. The only notable difference was an increase in protrusion head width compared to GFP controls. In contrast, the spines from Mtss1-S265D-S266D and Mtss1-S265A-S266A PCs showed a significantly decreased protrusion density and a slightly yet significantly reduced protrusion length suggesting that the changes in phosphorylation are not simply equivalent to a loss of function which was reported to produce an increase in protrusion length [26] (Fig. 5B-C). The protrusion head width was reduced compared to control GFP PCs and Mtss1-WT overexpression (Fig. 5D). These changes were also reflected in the reduced presence of mushroom spines, which are considered to be mature spines [34]. The phosphorylation mutants exhibited a reduction in mushroom spines compared to GFP controls, while Mtss1-WT overexpression showed a slight increase in these mature spines. In contrast, the phosphorylation mutants had a significantly increased percentage of stubby spines, which are considered to be less mature spines [35], and a slight increase in immature filopodia compared to control GFP PCs (Fig. 5E). Interestingly, the spines of PKCγ-A24E PCs present a phenotype similar to those of PCs with Mtss1 phospho-mutations. PKCγ-A24E PC spines show reduced protrusion density, length, and head width, a decreased percentage of mushroom spines, and an increased percentage of stubby spines compared to control-GFP PCs. The only significant difference between the spines of PKCγ-A24E PCs and those of Mtss1 phosphorylation mutants is the increased percentage of filopodia spines in PKCγ-A24E PCs compared to control-GFP and Mtss1-WT PC spines (Fig. 5A-E). These results show that Mtss1 phosphorylation is involved in the development of dendritic spines of PCs, a process also disrupted in PKCγ-A24E PCs.

**Fig. 6:**
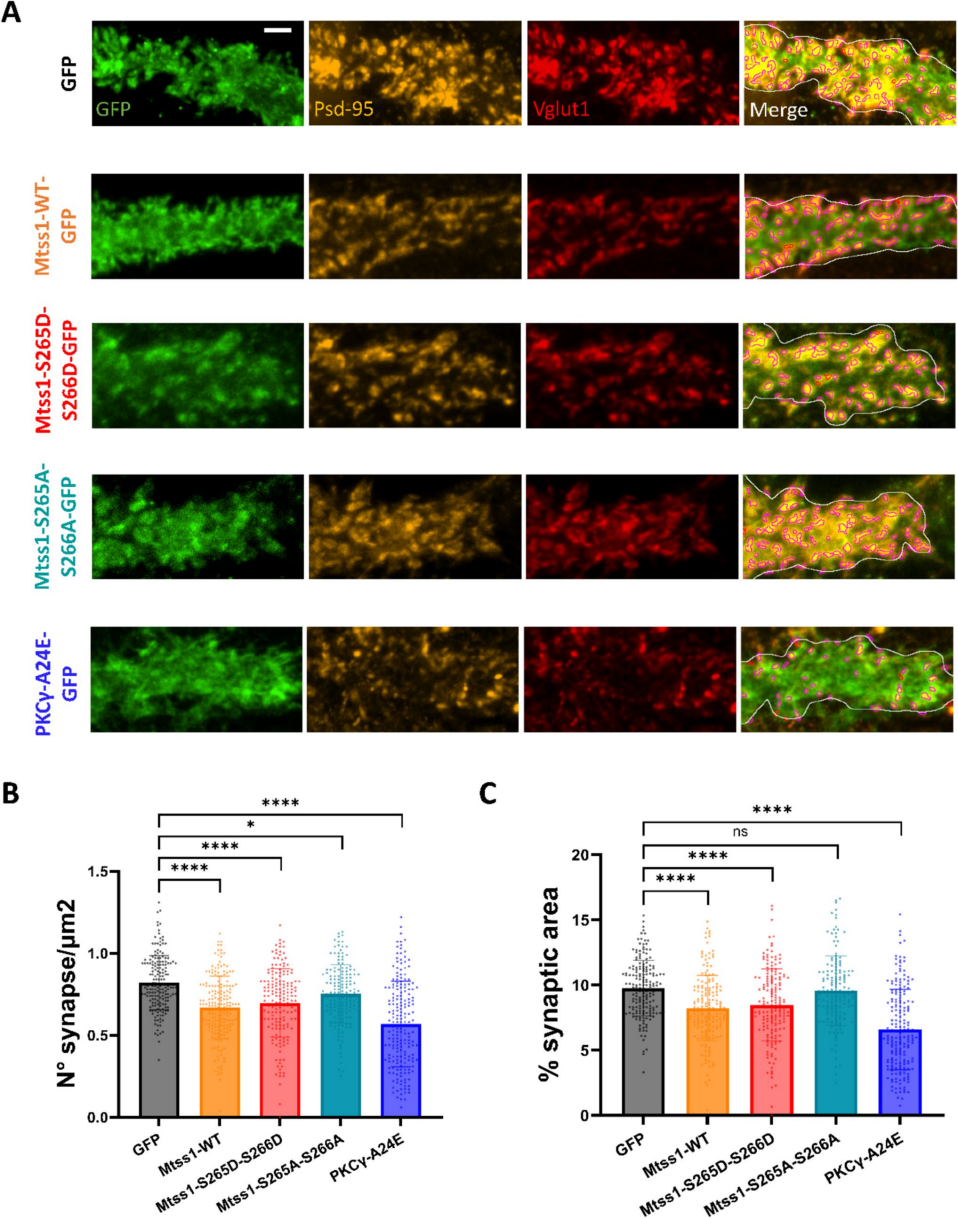
PC synapse formation is regulated by Mtss1 phosphorylation. (**A**) Representative images of PC dendritic segments for synapse quantification comparing GFP, Mtss1-WT-GFP, Mtss1-S265D-S266D-GFP, Mtss1-S265A-S266A, and PKCγ-A24E. GFP points dendritic segments of transfected PCs (green), PSD-95 stands for a post-synaptic marker (yellow), and VGLUT1 is a pre-synaptic marker (red). The merge shows the dots (pink) of detected overlaps between pre- and post-synaptic markers. Scale bar = 2 μm. (**B**) Number of mature synapses per μm^2^ and (**C**) percentage of synaptic area at each studied condition. Brown-Forsythe and Welch ANOVA test with post-hoc Dunnett comparisons test and Kruskal–Wallis test with post-hoc Dunn comparisons test; * = p-value < 0,05; ** = p-value < 0,01; *** = P-value < 0,001; **** = p-value < 0,0001. Error bars indicate SD. GFP N = 27 cells, n = 183 dendrites; Mtss1-WT N = 31 cells, n = 184 dendrites; Mtss1-S265D-S266D N = 32 cells, n = 173 dendrites; Mtss1-S265A-S266A N = 34 cells, n = 155 dendrites; PKCγ-A24E N = 40 cells, n = 196 dendrites. Data results from 5–8 dendritic segments/cell; 4–10 cells/culture; 3–5 independent cultures per condition

We then examined whether the changes of spine morphology induced by Mtss1 phosphorylation would also affect synapse formation. To this end, we defined active synapses as dendritic spine areas positive for the pre-synaptic marker, VGLUT1, and the post-synaptic marker, PSD-95, as previously described [36, 37]. We quantified the number of active synapses in PCs expressing the different Mtss1 phosphorylation constructs and we found that PCs with Mtss1-WT overexpression, Mtss1-S265D-S266D and Mtss1-S265A-S266A mutations showed a significantly decreased number of active synapses per µm^2^. Additionally, PCs with Mtss1-WT overexpression and Mtss1-S265D-S266D mutations showed a significantly reduced percentage of synaptic area (Fig. 6A-C). These findings suggest that both Mtss1-WT overexpression and Mtss1 phosphorylation result in less efficient synaptic formation, a process closely associated with alterations in spine morphology. An even stronger decrease of active synapses was found in PCs with PKCγ-A24E mutations suggesting that although Mtss1 is involved in the SCA14 phenotype, additional molecules/processes might contribute to the A24E phenotype.

### Partial Rescue of Mtss1-phosphorylation Related PC Dendritic Reduction Via Blockade of the Arp2/3 Complex

The Mtss1 and Arp2/3 complex are promoting the formation of dynamic lamellipodia-like actin protrusions contributing to the integrity of cell–cell junctions of epithelial cells [38]. It was previously demonstrated that the cooperation of Mtss1 and Arp 2/3 complex at the initial site of dendritic protrusions is crucial for the regulation of proper actin dynamics involved in spine development [25, 26]. Orchestrating actin dynamics for the formation of filopodia and lamellipodia is crucial not only for spine formation but also for the formation of neurites and the development of dendritic trees [39]. We investigated whether the reduction of PC dendritic complexity due to Mtss1 phosphorylation is regulated by Arp2/3 complex and actin dynamics, and whether PKCγ may serve as an upstream regulator of this molecular mechanism.

**Fig. 7:**
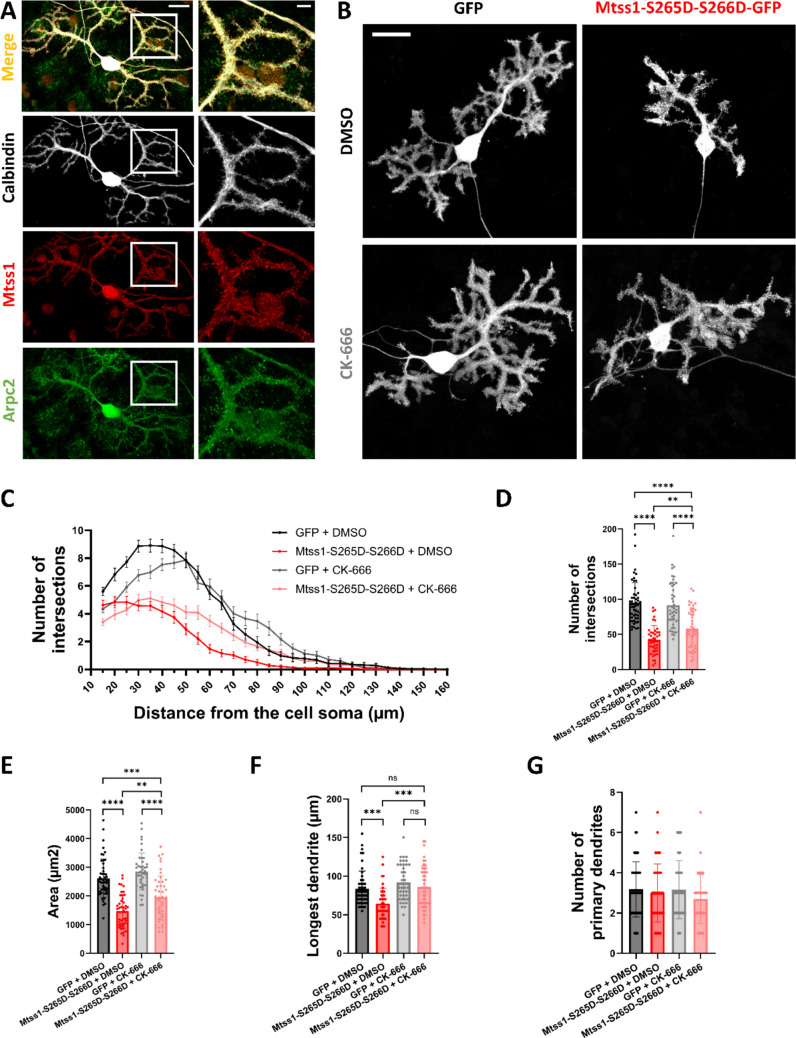
PC dendritic disruption caused by Mtss1 phosphorylation is partially regulated via the Arp2/3 complex. (**A**) Immunofluorescence staining showing colocalization of Mtss1 (red) and Arpc2 (green) in PCs (calbindin, white) of DCCs. Magnification shows colocalization in PC dendrites and PC spines. Scale bar = 20/5 μm. (**B**) Representative images of PCs at DIV21, transfected with GFP (Control) or Mtss1-S265D-S266D-GFP, and biochemically treated with DMSO (Control) or CK-666 (Arp2/3 inhibitor). Fluorescence detection: GFP. Scale bar = 20 μm. (**C**) Sholl analysis (data represented as mean ± SEM), (**D**) total number of intersections, (**E**) area, (**F**) longest dendrite, and (**G**) number of primary dendrites of PCs transfected with GFP + DMSO (n = 50 cells), Mtss1-S265D-S266D + DMSO (n = 43 cells), GFP + CK-666 (n = 44 cells), and Mtss1-S265D-S266D + CK-666 (n = 47 cells). Brown-Forsythe and Welch ANOVA test with post-hoc Dunnett comparisons test and Kruskal–Wallis test with post-hoc Dunn comparisons test; * = p-value < 0,05; ** = p-value < 0,01; *** = P-value < 0,001; **** = p-value < 0,0001. Error bars (**D-G**) indicate SD. Data produced in 3–5 independent cultures per condition

We first confirmed the co-localization of Mtss1 and Arp2/3 in PC dendrites of DCCs (Fig. 7A). Next, we transfected PCs with either control GFP or Mtss1-S265D-S266D mutations in DCCs in vitro and the cultures were treated with an Arp2/3 complex inhibitor, CK-666, comparing them to DMSO-treated controls. Sholl analysis showed no significant changes in the total number of intersections, area, or longest dendrite between control GFP PCs treated with 20 µM of CK-666 and GFP PCs treated with DMSO. In contrast, Mtss1-S265D-S266D PCs treated with 20 µM of CK-666 had a significantly increased number of intersections, with an increased area and longest dendrite compared to Mtss1-S265D-S266D PCs treated with DMSO. It is important to note that this rescuing effect of CK-666 was incomplete, and these parameters were still significantly reduced in Mtss1-S265D-S266D PCs treated with 20 µM of CK-666 compared to GFP PCs treated with DMSO (Fig. 7 A-G). Nevertheless, our findings suggest that PKCγ regulates Mtss1-Arp2/3 complex interactions in the development of PC dendrites, although additional factors also contribute to the proper PC dendritic development.

Considering the partial rescue observed in Mtss1 phospho-mimetic PCs, we examined whether similar improvements in PC dendritic development could be seen in PKCγ-A24E transfected PCs under CK-666 treatment. However, no significant changes were observed in the morphological parameters of PKCγ-A24E PCs treated with 20 µM CK-666 compared to PKCγ-A24E PCs treated with control DMSO (Fig. S7 A-E). Altogether, these results indicate that the loss of PC dendritic complexity mediated by Mtss1 phosphorylation is partially regulated through Arp2/3 complex although Arp 2/3 inhibition is not sufficient to rescue the PKCγ-A24E phenotype.

## Discussion

This study provides novel insights into the role of Mtss1 during PC dendritic development and its connection to the PKCγ molecular pathway and cytoskeleton signalling in SCA14. Our findings reveal that PKCγ-mediated phosphorylation of Mtss1 at S265 and S266 regulates PC dendritic outgrowth, spine development, and synapse formation. Our finding that these effects are dependent on increased phosphorylation and are not observed after overexpression of WT protein demonstrates that Mtss1 phosphorylation is an important downstream mediator of PKCγ function linking increased PKCγ activity to cytoskeletal changes. In line with this finding, the disruption of PC dendritic development through increased phosphorylation of Mtss1 is reversible, at least partially, by inhibition of Arp 2/3 complex.

### Mtss1 Characterization in PC Development and SCA

Mtss1 was reported to be an important determinant of PC dendritic development. In Mtss1-deficient mice, the PC dendritic arbour is greatly reduced and Mtss1 was shown to interact with the actin cytoskeleton in dendrites via the Arp2/3 complex and the formin DAAM1 [26]. In SCA1 and SCA2 the expression levels of Mtss1 were found to be greatly reduced suggesting a role of Mtss1 as an ataxia gene linking multiple SCAs [28]. Interestingly, both loss and gain of Mtss1 have been associated with disease, highlighting the importance of maintaining optimal Mtss1 levels [40, 41]. Our findings demonstrate that Mtss1 protein levels in the cerebellum of three-week-old PKCγ-A24E mice (SCA14 mouse model) are significantly increased compared to WT. Age-related changes in Mtss1 protein levels have attracted the interest of several researchers. Previous studies have demonstrated that Mtss1 expression is developmentally regulated in the murine cerebellum, suggesting a role in cytoskeletal dynamics of developing cerebellar neurons [42]. Moreover, Mtss1 has been shown to promote the maturation and maintenance of cerebellar neurons through splice variant-specific effects [43]. Consistent with this, our results indicate that Mtss1 expression reaches its highest level in the cerebellum at P14, emphasizing its critical role in PC development.

Using phospho-proteomics, we identified two Mtss1 phospho-sites (S265 and S266) that display increased phosphorylation levels in PKCγ-A24E mice compared to WTs. Due to the lack of phospho-specific antibodies for Mtss1, its increased phosphorylation in cells with increased PKCγ activity was indirectly validated by detecting elevated phospho-serine PKC substrate levels at the Mtss1 protein size and by demonstrating an interaction between PKCγ and Mtss1 through immunoprecipitation. Thus, both the expression and phosphorylation of Mtss1 were changed in PKCγ-A24E mice suggesting an important role for PC dendritic development.

### Mtss1 Phosphorylation at S265-S266 Regulates PC Dendritic Outgrowth

Mtss1 plays a role in the branching of neurites, neuronal morphogenesis, organization of the actin cytoskeleton, and filopodia formation. The Mtss1-KO resulted in a decreased PC dendritic complexity [26, 28], suggesting that an Mtss1 overexpression might enhance PC dendritic growth. When we overexpressed Mtss1-WT in PCs of DCCs, PC dendritic development was intact and similar to GFP-control PCs. In contrast, transfection of PCs with constructs either mimicking the phosphorylated state (S265D-S266D) or preventing phosphorylation (S265A-S266A) at the two phospho-sites identified in phospho-proteomics both resulted in impaired PC dendritic arborization highlighting the importance of Mtss1 phosphorylation. Many proteins require precise phosphorylation for interactions with actin regulators [44]. Both excessive and absent phosphorylation may disrupt these interactions triggering similar defects on dendritic spine morphology [45]. Our results suggest that Mtss1 phosphorylation modulates its interactions and activity to regulate actin dynamics and dendritic growth, emphasizing the importance of optimal Mtss1 phosphorylation for PC dendritic development.

The Mtss1 phospho-mimetic mutations mimicked the phenotype present in our PKCγ-A24E SCA14 mouse model in which Mtss1 phosphorylation is increased at these two sites but did not reproduce it completely. Interestingly, in PCs transfected with PKCγ-A24E the number of primary dendrites coming directly from the cell soma is strongly increased. Although the disruption of PC dendritic trees in PKCγ-A24E PCs was previously reported [18] it remained unclear at which developmental stage PCs began to be affected. Here we show that the morphology of PKCγ-A24E transfected PCs in DCCs does not progress from DIV10 to DIV21 suggesting that these PCs get developmentally blocked in the stellate stage with an inhibition of neurite retraction. In contrast, PCs with Mtss1 phospho-mimetic and phospho-defective mutations present an intact neurite retraction suggesting that the disruption of the dendritic tree is caused by either a partial impairment in branch initiation or an increased branch retraction [46]. During PC development, neurite retraction is a process that precedes dendrite elongation and the initiation of dendritic branches near the growing terminals [47]. Selective inhibition of neurite retraction in vitro, while preserving dendrite elongation and the balanced dynamics of branch initiation and contact-induced branch retraction, could potentially enhance the dendritic complexity of PCs [48]. For PKCγ-A24E transfected PCs to be blocked in the stellate stage, both the inhibition of neurite retraction and a disruption of dendrite elongation are likely to be involved.

Mtss1-KO PCs have been reported to exhibit reduced dendritic trees due to increased contact-dependent retraction and to have longer dendritic protrusions [26]. Our Mtss1 phosphorylation mutants, however, show decreased protrusion length, indicating that the observed morphological changes differ from those of Mtss1-KO PCs. Additionally, considering that Mtss1 is linked to the Arp2/3 complex [26, 49], which is involved in branch initiation, the reduced dendritic arborization observed in PCs with Mtss1 phospho-mutants could result from decreased branch initiation. However, based on our results, it remains unclear whether the disruption in PCs with altered Mtss1 phosphorylation is due to decreased branch initiation or increased branch retraction. Nevertheless, we assume that the disruption of dendrite elongation in PCs with an altered Mtss1 phosphorylation contributes to the PKCγ-A24E phenotype. PKCγ-A24E transfected PCs, in addition, display defects in neurite retraction blocking them in the stellate state suggesting the implication of additional altered molecular processes.

### Mtss1 Phosphorylation at S265-S266 Regulates PC Spine Development and Synapse Formation

Among the various functions of PKCγ during PC development is its role in regulating spine development [50, 51]. In parallel, previous studies have associated Mtss1 with spine morphology, indicating that it promotes dendritic spine initiation [52] and regulates protrusion length in a dose-dependent manner [26, 53]. Interestingly, PCs with Mtss1-WT overexpression did not show changes in spine length, although they exhibited an increased head width compared to GFP controls. However, altering PKCγ-mediated phosphorylation of Mtss1 at S265-S266 leads to a decreased spine density and length, and a decreased protrusion head width, similar to what is observed in PKCγ-A24E transfected PCs. These findings suggest that the role of PKCγ in spine development is mediated, at least in part, through the phosphorylation of Mtss1 at S265-S266, and that Mtss1 requires an optimal and dynamic phosphorylation at S265-S266 to refine spine morphology.

In most of the existing ataxia models, PCs exhibit alterations in synaptic function which leads to the disruption of cerebellar circuits triggering ataxic symptoms [54]. PKCγ-A24E mice were previously shown to have a reduced climbing fiber innervation in vivo [18]. Consistent with this, our results suggest a reduction in the number of synapses in PKCγ-A24E transfected PCs in vitro. A similar decrease was also observed in PCs transfected with Mtss1 phospho-mimetic and phospho-defective mutations, indicating the importance of an optimal and dynamic PKCγ-mediated phosphorylation of Mtss1. Although this method has been used to assess synapse formation [36, 37], it lacks the resolution of electron microscopy and therefore cannot provide definitive confirmation. Still, the observed differences in the expression of both pre- and post-synaptic markers (VGLUT1 and PSD-95) are consistent with synaptic reduction.

According to our results, PCs with Mtss1-WT overexpression also exhibit a reduction in the number of synapses without major changes in spine morphology. Both synaptic activity and spine morphology are closely linked in a complex bidirectional relationship [55, 56], with synaptic activity influencing dendritic spine morphology [57]. Although synaptic disruption in Purkinje cells has been reported to precede morphological alterations [58], evidence showing no changes in PC spine morphology during long-term depression [59] suggests that the relationship between synapses and spine morphology may be influenced by the type of synaptic plasticity involved. We speculate that in our experiments, synaptic processes may be affected in Mtss1-WT PCs before major changes in spine morphology occur, rather than changes in spine morphology triggering synaptic disruption. However, further experiments are required to clarify this interplay.

### PC Dendritic Disruption Mediated by Mtss1 Phosphorylation is Partially Regulated Via the Arp2/3 Complex

The molecular interaction between Mtss1 and the Arp2/3 complex to regulate actin dynamics has been previously studied [26, 60], and Mtss1 was shown to promote the outgrowth of dendritic vs axonal processes in dissociated neuronal cultures [61]. The importance of Arp2/3 for filopodia formation, growth cone extension, and neurogenesis is well documented [62, 63], and inhibition of Arp2/3 function disrupts neuronal morphogenesis [64, 65]. Interestingly, in our experiments, treatment with the Arp2/3 inhibitor CK-666 did not negatively affect dendritic morphology in GFP control PCs. This could be due to the relatively low concentration of CK-666 used in order to avoid toxicity in PCs or the timing of the CK-666 treatment, which only began at DIV14-15, i.e. after an early developmental phase where Arp2/3 complex is critical for PC maturation [65].

The Arp2/3 complex can be regulated by PKC-mediated phosphorylation of intermediate actin-binding proteins like Coronin 1B [66]. The partial rescue of dendritic complexity in Mtss1 phospho-mimetic PCs treated with CK-666 suggests that PKCγ acts as an upstream regulator of the Mtss1-Arp2/3 molecular pathway in PC development. Under normal conditions, Mtss1 positively regulates Arp2/3 activity in PC dendrites [26]. Based on our findings, we suggest that dysregulated Mtss1 phosphorylation leads to excessive Arp2/3 activation, disrupting actin dynamics and affecting the formation of filopodia and lamellipodia, processes essential for proper PC dendritic development [67]. We propose that also an excess of Arp2/3 and the absence of dynamically promoting and inhibiting Arp 2/3 function is disadvantageous for dendrite growth, highlighting the need for precise regulation of the Arp2/3 complex during neurodevelopment. However, the incomplete rescue in Mtss1 phospho-mimetic PCs and the lack of a rescuing effect in PKCγ-A24E PCs treated with CK-666 suggest that additional factors might be involved in regulating actin dynamics during PC development.

### PKCγ, Mtss1, PC Dendritic Development and Pathogenesis of SCA14

**Fig. 8:**
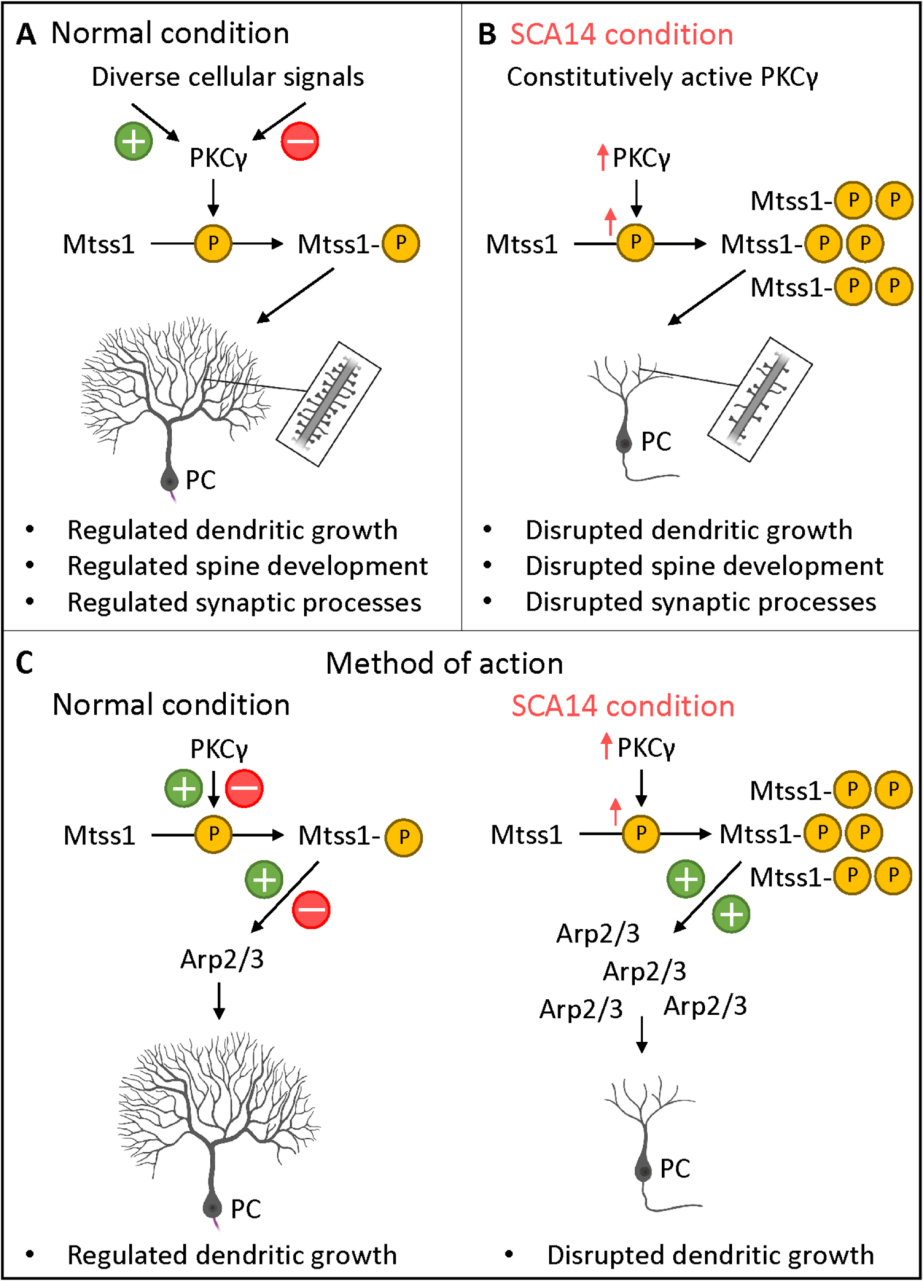
Molecular model of disease. (**A**) Under normal conditions, PKCγ phosphorylates Mtss1 resulting in the regulation of dendritic growth, spine development, and synaptic processes of PCs. (**B**) Under our SCA14 condition, an overactivation of PKCγ leads to the increased phosphorylation of Mtss1 (specifically in the phospho-sites S265 and S266) which triggers the disruption of PKCγ-related processes such as dendritic growth, spine development and synaptic processes of PCs, partially mimicking the SCA14 phenotype. (**C**) Method of action. The regulation of PC development related to PKCγ-Mtss1 axis is partially regulated via the Arp2/3 complex. Under normal conditions, Mtss1 is positively modulating Arp2/3 complex activity in PC dendrites. We hypothesize that under the SCA14 condition, the altered Mtss1 phosphorylation leads to an overactivation of Arp2/3 which might be involved in some PC developmental processes such as PC dendritic growth

In our laboratory, we have previously identified CRMP2 and Stk-17b as phosphorylation targets of PKCγ mediating the effects of increased PKC activity present in mouse models of SCA14 and in other forms of SCA [15–17, 68]. We now have identified Mtss1 phosphorylation as novel mediator of PKCγ activity. We propose that Mtss1 is a phosphorylation target of PKCγ which links PKC activity to the modulation of the actin cytoskeleton and thus to dendritic morphology. We have shown that a dysregulation of Mtss1 by increased PKCγ activity or by a mutation of the S265-S266 phospho-sites interferes with PC dendritic development, spine morphology and synapse formation. We could identify PKCγ as an upstream regulator of Mtss1 activity and have shown that proper Mtss1 regulation is crucial for the development of PC morphology. The function of Mtss1 in PC development critically depends upon precise and dynamic phosphorylation at the S265-S266 phospho-sites modulating the Arp2/3 complex and thus regulating actin dynamics. A disruption of the critical balance of PKCγ activation and inactivation is interfering with the proper regulation of actin dynamics and thus the regulation of process outgrowth and retraction, eventually resulting in compromised dendritic development and synaptic dysfunction. Our current model of how a constitutive activation of PKCγ interferes with the proper regulation of Mtss1 phosphorylation and cytoskeletal dynamics is depicted in Fig. 8.

We recognize that our findings are currently based on in vitro experiments only, and further studies are required to determine whether the marked phenotype shown in PCs with altered Mtss1 phosphorylation is also present in vivo. While our experiments focus on a SCA14 mouse model, the reported involvement of Mtss1 in other types of ataxia [28] suggests that the PKCγ-Mtss1 molecular mechanisms described here could be relevant for multiple SCAs. This highlights the importance of elucidating the molecular pathways underlying PC development as a foundation for future therapeutic strategies.

## Supplementary Information

Below is the link to the electronic supplementary material.

Supplementary file1 (PDF 15168 KB)

Supplementary file2 (PDF 2715 KB)

## Data Availability

Data will be made available upon request by the authors.

## References

[1] van der Heijden ME, Sillitoe RV (2020) Interactions between Purkinje cells and granule cells coordinate the development of functional cerebellar circuits. Neuroscience 462:4. 10.1016/J.NEUROSCIENCE.2020.06.010 32554107 10.1016/j.neuroscience.2020.06.010 PMC7736359

[2] Fernández Santoro EM, Karim A, Warnaar P et al (2024) Purkinje cell models: past, present and future. Front Comput Neurosci 18:1426653. 10.3389/FNCOM.2024.1426653/BIBTEX 39049990 10.3389/fncom.2024.1426653 PMC11266113

[3] Tanaka-Yamamoto K, Shakkottai V, Becker EBE et al (2020) Aberrant cerebellar circuitry in the spinocerebellar ataxias. Front Neurosci 14:707. 10.3389/FNINS.2020.00707 32765211 10.3389/fnins.2020.00707 PMC7378801

[4] Chen DH, Raskind WH, Bird TD (2012) Spinocerebellar ataxia type 14. In: Handbook of Clinical Neurology. Elsevier, pp 555–559 10.1016/B978-0-444-51892-7.00036-X 21827914

[5] Wong MMK, Hoekstra SD, Vowles J et al (2018) Neurodegeneration in SCA14 is associated with increased PKCγ kinase activity, mislocalization and aggregation. Acta Neuropathol Commun 6:99. 10.1186/s40478-018-0600-7 30249303 10.1186/s40478-018-0600-7 PMC6151931

[6] Sun R, Tang X, Cao X et al (2023) Novel mutation in exon11 of PRKCG (SCA14): A case report. Front Genet 14:1129988. 10.3389/FGENE.2023.1129988/BIBTEX 36968593 10.3389/fgene.2023.1129988 PMC10031122

[7] Verbeek DS, Knight MA, Harmison GG et al (2005) Protein kinase C gamma mutations in spinocerebellar ataxia 14 increase kinase activity and alter membrane targeting. Brain 128:436–442. 10.1093/brain/awh378 15618281 10.1093/brain/awh378

[8] Pilo CA, Baffi TR, Kornev AP et al (2022) Mutations in protein kinase Cγ promote spinocerebellar ataxia type 14 by impairing kinase autoinhibition. Sci Signal 15:1147. 10.1126/SCISIGNAL.ABK1147/SUPPL_FILE/SCISIGNAL.ABK1147_MDAR_REPRODUCIBILITY_CHECKLIST.PDF 10.1126/scisignal.abk1147 PMC9810342 36166510

[9] Verbeek DS, Goedhart J, Bruinsma L et al (2008) PKCγ mutations in spinocerebellar ataxia type 14 affect C1 domain accessibility and kinase activity leading to aberrant MAPK signaling. J Cell Sci 121:2339–2349. 10.1242/jcs.027698 18577575 10.1242/jcs.027698

[10] Shimobayashi E, Kapfhammer JP (2017) Increased biological activity of protein kinase C gamma is not required in Spinocerebellar ataxia 14. Mol Brain 10:1–11. 10.1186/S13041-017-0313-Z/FIGURES/5 28738819 10.1186/s13041-017-0313-z PMC5525338

[11] Ghanekar SD, Kuo SH, Staffetti JS, Zesiewicz TA (2022) Current and emerging treatment modalities for spinocerebellar ataxias. Expert Rev Neurother 22:101–114. 10.1080/14737175.2022.2029703 35081319 10.1080/14737175.2022.2029703 PMC9048095

[12] Shimobayashi E, Kapfhammer JP (2018) Calcium signaling, PKC gamma, IP3R1 and CAR8 link spinocerebellar ataxias and Purkinje cell dendritic development. Curr Neuropharmacol 16:151. 10.2174/1570159X15666170529104000 28554312 10.2174/1570159X15666170529104000 PMC5883377

[13] Lordén G, Newton AC (2021) Conventional protein kinase C in the brain: repurposing cancer drugs for neurodegenerative treatment? Neuronal Signal 5:NS20210036. 10.1042/NS20210036 34737895 10.1042/NS20210036 PMC8536831

[14] Schrenk K, Kapfhammer JP, Metzger F (2002) Altered dendritic development of cerebellar Purkinje cells in slice cultures from protein kinase Cγ-deficient mice. Neuroscience 110:675–689. 10.1016/S0306-4522(01)00559-0 11934475 10.1016/s0306-4522(01)00559-0

[15] Winkler SC, Shimobayashi E, Kapfhammer JP (2020) PKCγ-mediated phosphorylation of CRMP2 regulates dendritic outgrowth in cerebellar Purkinje cells. Mol Neurobiol 57:5150–5166. 10.1007/S12035-020-02038-6 32860158 10.1007/s12035-020-02038-6 PMC7541385

[16] Wu QW, Kapfhammer JP (2021) Modulation of increased mGluR1 signaling by RGS8 protects Purkinje cells from dendritic reduction and could be a common mechanism in diverse forms of spinocerebellar ataxia. Front Cell Dev Biol 8:569889. 10.3389/FCELL.2020.569889 33553137 10.3389/fcell.2020.569889 PMC7858651

[17] Wu QW, Kapfhammer JP (2021) Serine/threonine kinase 17b (STK17B) signalling regulates Purkinje cell dendritic development and is altered in multiple spinocerebellar ataxias. Eur J Neurosci 54:6673–6684. 10.1111/EJN.15465 34536317 10.1111/ejn.15465 PMC9292345

[18] Shimobayashi E, Kapfhammer JP (2021) A new mouse model related to SCA14 carrying a pseudosubstrate domain mutation in PKCγ shows perturbed Purkinje cell maturation and ataxic motor behavior. J Neurosci 41:2053. 10.1523/JNEUROSCI.1946-20.2021 33478986 10.1523/JNEUROSCI.1946-20.2021 PMC7939089

[19] Wang Y, Jia Z, Liang C, et al (2023) MTSS1 curtails lung adenocarcinoma immune evasion by promoting AIP4-mediated PD-L1 monoubiquitination and lysosomal degradation. Cell Discov 9:1–15. 10.1038/s41421-022-00507-x 10.1038/s41421-022-00507-x PMC9944270 36810288

[20] Wu M, Qiu Q, Zhou Q et al (2022) circFBXO7/miR-96-5p/MTSS1 axis is an important regulator in the Wnt signaling pathway in ovarian cancer. Mol Cancer 21:1–19. 10.1186/S12943-022-01611-Y/FIGURES/7 35768865 10.1186/s12943-022-01611-y PMC9241180

[21] Xie F, Ye L, Ta M, et al (2011) MTSS1: A multifunctional protein and its role in cancer invasion and metastasis. Front Biosci - Sch 3 S:621–631. 10.2741/S175/PDF 10.2741/s175 21196400

[22] Liu K, Jiao XD, Hao JL et al (2019) MTSS1 inhibits metastatic potential and induces G2/M phase cell cycle arrest in gastric cancer. Onco Targets Ther 12:5143. 10.2147/OTT.S203165 31303767 10.2147/OTT.S203165 PMC6612291

[23] Yang C, Hoelzle M, Disanza A et al (2009) Coordination of membrane and actin cytoskeleton dynamics during filopodia protrusion. PLoS One 4:e5678. 10.1371/JOURNAL.PONE.0005678 19479071 10.1371/journal.pone.0005678 PMC2682576

[24] Matskova L, Zheng S, Kashuba E et al (2024) MTSS1: beyond the integration of actin and membrane dynamics. Cell Mol Life Sci 81:472. 10.1007/S00018-024-05511-W 39625546 10.1007/s00018-024-05511-w PMC11615175

[25] Parker SS, Ly KT, Grant AD et al (2023) EVL and MIM/MTSS1 regulate actin cytoskeletal remodeling to promote dendritic filopodia in neurons. J Cell Biol 222:e202106081. 10.1083/JCB.202106081 36828364 10.1083/jcb.202106081 PMC9998662

[26] Kawabata Galbraith K, Fujishima K, Mizuno H et al (2018) MTSS1 regulation of actin-nucleating formin DAAM1 in dendritic filopodia determines final dendritic configuration of Purkinje cells. Cell Rep 24:95-106.e9. 10.1016/J.CELREP.2018.06.013 29972794 10.1016/j.celrep.2018.06.013

[27] Egorova PA, Bezprozvanny IB (2019) Molecular mechanisms and therapeutics for spinocerebellar ataxia type 2. Neurotherapeutics 16:1050–1073. 10.1007/S13311-019-00777-6 31435879 10.1007/s13311-019-00777-6 PMC6985344

[28] Brown AS, Meera P, Altindag B et al (2018) MTSS1/Src family kinase dysregulation underlies multiple inherited ataxias. Proc Natl Acad Sci U S A 115:E12407–E12416. 10.1073/PNAS.1816177115 30530649 10.1073/pnas.1816177115 PMC6310854

[29] Shimobayashi E, Wagner W, Kapfhammer JP (2016) Carbonic anhydrase 8 expression in Purkinje cells is controlled by PKCγ activity and regulates Purkinje cell dendritic growth. Mol Neurobiol 53:5149–5160. 10.1007/S12035-015-9444-3 26399641 10.1007/s12035-015-9444-3

[30] Wagner W, McCroskery S, Hammer JA (2011) An efficient method for the long-term and specific expression of exogenous cDNAs in cultured Purkinje neurons. J Neurosci Methods 200:95–105. 10.1016/J.JNEUMETH.2011.06.006 21708190 10.1016/j.jneumeth.2011.06.006 PMC3407467

[31] Ferreira TA, Blackman AV, Oyrer J et al (2014) Neuronal morphometry directly from bitmap images. Nat Methods. 10.1038/nmeth.3125 25264773 10.1038/nmeth.3125 PMC5271921

[32] Lin L, Lo LHY, Lyu Q, Lai KO (2017) Determination of dendritic spine morphology by the striatin scaffold protein STRN4 through interaction with the phosphatase PP2A. J Biol Chem 292:9451–9464. 10.1074/JBC.M116.772442 28442576 10.1074/jbc.M116.772442 PMC5465475

[33] Schindelin J, Arganda-Carreras I, Frise E, et al (2012) Fiji: an open-source platform for biological-image analysis. Nat Methods 9:676–682. 10.1038/nmeth.2019 10.1038/nmeth.2019 PMC3855844 22743772

[34] Alimohamadi H, Bell MK, Halpain S, Rangamani P (2021) Mechanical principles governing the shapes of dendritic spines. Front Physiol 12:657074. 10.3389/FPHYS.2021.657074 34220531 10.3389/fphys.2021.657074 PMC8242199

[35] Pchitskaya E, Bezprozvanny I (2020) Dendritic spines shape analysis—classification or clusterization? Perspective. Front Synaptic Neurosci 12:556375. 10.3389/FNSYN.2020.00031 10.3389/fnsyn.2020.00031 PMC7561369 33117142

[36] Verstraelen P, Van Dyck M, Verschuuren M et al (2018) Image-based profiling of synaptic connectivity in primary neuronal cell culture. Front Neurosci 12:389. 10.3389/FNINS.2018.00389 29997468 10.3389/fnins.2018.00389 PMC6028601

[37] Savage JT, Ramirez JJ, Risher WC et al (2024) SynBot is an open-source image analysis software for automated quantification of synapses. Cell Rep Methods 4:100861. 10.1016/j.crmeth.2024.100861 39255792 10.1016/j.crmeth.2024.100861 PMC11440803

[38] Senju Y, Mushtaq T, Vihinen H, et al (2023) Actin-rich lamellipodia-like protrusions contribute to the integrity of epithelial cell–cell junctions. J Biol Chem 299. 10.1016/J.JBC.2023.104571/ATTACHMENT/97C7A520-3480-4FC8-828D-436AE384F055/MMC17.PDF 10.1016/j.jbc.2023.104571 PMC10173786 36871754

[39] Flynn KC (2013) The cytoskeleton and neurite initiation. Bioarchitecture 3:86. 10.4161/BIOA.26259 24002528 10.4161/bioa.26259 PMC4201609

[40] Macoska YGLJA, Korenchuk S, Pienta KJ (2002) MIM, a potential metastasis suppressor gene in bladder cancer. Neoplasia 4:291–294. 10.1038/SJ.NEO.7900231 12082544 10.1038/sj.neo.7900231 PMC1531703

[41] Callahan CA, Ofstad T, Horng L et al (2004) MIM/BEG4, a sonic hedgehog-responsive gene that potentiates Gli-dependent transcription. Genes Dev 18:2724–2729. 10.1101/GAD.1221804 15545630 10.1101/gad.1221804 PMC528890

[42] Glassmann A, Molly S, Surchev L et al (2007) Developmental expression and differentiation-related neuron-specific splicing of metastasis suppressor 1 (Mtss1) in normal and transformed cerebellar cells. BMC Dev Biol 7:1–15. 10.1186/1471-213X-7-111 17925019 10.1186/1471-213X-7-111 PMC2194783

[43] Sistig T, Lang F, Wrobel S et al (2017) MTSS1 promotes maturation and maintenance of cerebellar neurons via splice variant-specific effects. Brain Struct Funct 222:2787–2805. 10.1007/S00429-017-1372-8 28214917 10.1007/s00429-017-1372-8

[44] Sun J, Zhong X, Fu X et al (2022) The actin regulators involved in the function and related diseases of lymphocytes. Front Immunol 13:799309. 10.3389/FIMMU.2022.799309 35371070 10.3389/fimmu.2022.799309 PMC8965893

[45] Cornelius J, Haak S, Rothkegel M et al (2023) Phosphorylation of the actin-binding protein profilin2a at S137 modulates bidirectional structural plasticity at dendritic spines. Front Cell Dev Biol 11:1107380. 10.3389/FCELL.2023.1107380 36875774 10.3389/fcell.2023.1107380 PMC9975505

[46] Ouzounidis VR, Prevo B, Cheerambathur DK (2023) Sculpting the dendritic landscape: actin, microtubules, and the art of arborization. Curr Opin Cell Biol 84:102214. 10.1016/J.CEB.2023.102214 37544207 10.1016/j.ceb.2023.102214

[47] Sotelo C, Dusart I (2009) Intrinsic versus extrinsic determinants during the development of Purkinje cell dendrites. Neuroscience 162:589–600. 10.1016/J.NEUROSCIENCE.2008.12.035 19166910 10.1016/j.neuroscience.2008.12.035

[48] Fujishima K, Horie R, Mochizuki A, Kengaku M (2012) Principles of branch dynamics governing shape characteristics of cerebellar Purkinje cell dendrites. Dev 139:3442–3455. 10.1242/DEV.081315/-/DC1 10.1242/dev.081315 PMC3491647 22912417

[49] Lin J, Liu J, Wang Y et al (2005) Differential regulation of cortactin and N-WASP-mediated actin polymerization by missing in metastasis (MIM) protein. Oncogene 24(12):2059–2066. 10.1038/sj.onc.1208412 15688017 10.1038/sj.onc.1208412

[50] Seki T, Shimahara T, Yamamoto K et al (2009) Mutant γPKC found in spinocerebellar ataxia type 14 induces aggregate-independent maldevelopment of dendrites in primary cultured Purkinje cells. Neurobiol Dis 33:260–273. 10.1016/J.NBD.2008.10.013 19041943 10.1016/j.nbd.2008.10.013

[51] Sziber Z, Torrents-Solé P, Kovacevic A, Kapfhammer JP (2025) Protein kinase C gamma regulates Purkinje cell dendritic spine development in a mouse model of spinocerebellar ataxia. Exp Neurol 393:115377. 10.1016/J.EXPNEUROL.2025.115377 40675361 10.1016/j.expneurol.2025.115377

[52] Saarikangas J, Kourdougli N, Senju Y et al (2015) Mim-induced membrane bending promotes dendritic spine initiation. Dev Cell 33:644–659. 10.1016/J.DEVCEL.2015.04.014 26051541 10.1016/j.devcel.2015.04.014

[53] Khanal P, Hotulainen P (2021) Dendritic spine initiation in brain development, learning and diseases and impact of BAR-domain proteins. Cells 10(9):2392. 10.3390/CELLS10092392 34572042 10.3390/cells10092392 PMC8468246

[54] Hoxha E, Balbo I, Miniaci MC, Tempia F (2018) Purkinje cell signaling deficits in animal models of ataxia. Front Synaptic Neurosci 10:6. 10.3389/FNSYN.2018.00006 29760657 10.3389/fnsyn.2018.00006 PMC5937225

[55] Fan WJ, Yan MC, Wang L et al (2018) Synaptic aging disrupts synaptic morphology and function in cerebellar Purkinje cells. Neural Regen Res 13:1019. 10.4103/1673-5374.233445 29926829 10.4103/1673-5374.233445 PMC6022458

[56] Ammassari-Teule M (2020) Early-occurring dendritic spines alterations in mouse models of Alzheimer’s disease inform on primary causes of neurodegeneration. Front Synaptic Neurosci 12:566615. 10.3389/FNSYN.2020.566615 33013348 10.3389/fnsyn.2020.566615 PMC7511703

[57] Verpelli C, Piccoli G, Zibetti C et al (2010) Synaptic activity controls dendritic spine morphology by modulating eEF2-dependent BDNF synthesis. J Neurosci 30:5830–5842. 10.1523/JNEUROSCI.0119-10.2010 20427644 10.1523/JNEUROSCI.0119-10.2010 PMC6632604

[58] Wulff P, Schonewille M, Renzi M et al (2009) Synaptic inhibition of Purkinje cells mediates consolidation of vestibulo-cerebellar motor learning. Nat Neurosci 12:1042. 10.1038/NN.2348 19578381 10.1038/nn.2348 PMC2718327

[59] Sdrulla AD, Linden DJ (2007) Double dissociation between long-term depression and dendritic spine morphology in cerebellar Purkinje cells. Nat Neurosci. 10.1038/nn1889 10.1038/nn1889 17435753

[60] Podieh F, Overboom MC, Knol JC et al (2024) AAMP and MTSS1 are novel negative regulators of endothelial barrier function identified in a proteomics screen. Cells 13:1609. 10.3390/CELLS13191609/S1 39404373 10.3390/cells13191609 PMC11476176

[61] Yu J, Lin S, Wang M et al (2016) Metastasis suppressor 1 regulates neurite outgrowth in primary neuron cultures. Neuroscience 333:123–131. 10.1016/J.NEUROSCIENCE.2016.07.002 27401056 10.1016/j.neuroscience.2016.07.002

[62] Pinyol R, Haeckel A, Ritter A et al (2007) Regulation of N-WASP and the Arp2/3 complex by Abp1 controls neuronal morphology. PLoS One 2:e400. 10.1371/JOURNAL.PONE.0000400 17476322 10.1371/journal.pone.0000400 PMC1852583

[63] Korobova F, Svitkina T (2008) Arp2/3 complex is important for filopodia formation, growth cone motility, and neuritogenesis in neuronal cells. Mol Biol Cell 19(4):1561–1574. 10.1091/mbc.e07-09-0964 18256280 10.1091/mbc.E07-09-0964 PMC2291425

[64] Chou F-S, Wang P-S (2016) The Arp2/3 complex is essential at multiple stages of neural development. Neurogenesis 3:e1261653. 10.1080/23262133.2016.1261653 28405589 10.1080/23262133.2016.1261653 PMC5384616

[65] Hasegawa K, Matsui TK, Kondo J, Kuwako KI (2022) N-WASP-Arp2/3 signaling controls multiple steps of dendrite maturation in Purkinje cells in vivo. Dev 149. 10.1242/DEV.201214/VIDEO-3 10.1242/dev.201214 36469048

[66] Cai L, Holoweckyj N, Schaller MD, Bear JE (2005) Phosphorylation of coronin 1B by protein kinase C regulates interaction with Arp2/3 and cell motility. J Biol Chem 280:31913–31923. 10.1074/jbc.M504146200 16027158 10.1074/jbc.M504146200

[67] He Y, Ren Y, Wu B et al (2015) Src and cortactin promote lamellipodia protrusion and filopodia formation and stability in growth cones. Mol Biol Cell 26:3229. 10.1091/mbc.e15-03-0142 26224308 10.1091/mbc.E15-03-0142 PMC4569314

[68] Kapfhammer JP, Shimobayashi E (2023) Viewpoint: spinocerebellar ataxias as diseases of Purkinje cell dysfunction rather than Purkinje cell loss. Front Mol Neurosci 16:1182431. 10.3389/FNMOL.2023.1182431 37426070 10.3389/fnmol.2023.1182431 PMC10323145

