# Supplementary material for: PKCγ-mediated Phosphorylation of Mtss1 Regulates the Dendritic Outgrowth and Spine Development of Cerebellar Purkinje Cells
Source: Mol Neurobiol. 2025 Nov 25;63(1):168. doi: 10.1007/s12035-025-05526-9 (PMC12647342; doi:10.1007/s12035-025-05526-9)
Supplement: Supplementary file 1 — Supplementary file1 (PDF 15168 KB) [file 12035_2025_5526_MOESM1_ESM.pdf]

Supplementary Material

PKCγ-mediated phosphorylation of Mtss1 regulates the dendritic outgrowth and spine development of cerebellar Purkinje cells

Molecular Neurobiology

Paula Torrents-Solé[1#], Zsófia Sziber[1], Etsuko Shimobayashi[1*], Josef P Kapfhammer[1].

[1]Anatomical Institute, Department of Biomedicine, University of Basel, Basel CH-4056, Switzerland.

[*]Current address: Takeda Pharmaceuticals, Yokohama, Japan.

[#]Corresponding author: paula.torrentssole@unibas.ch

Fig. S1

A

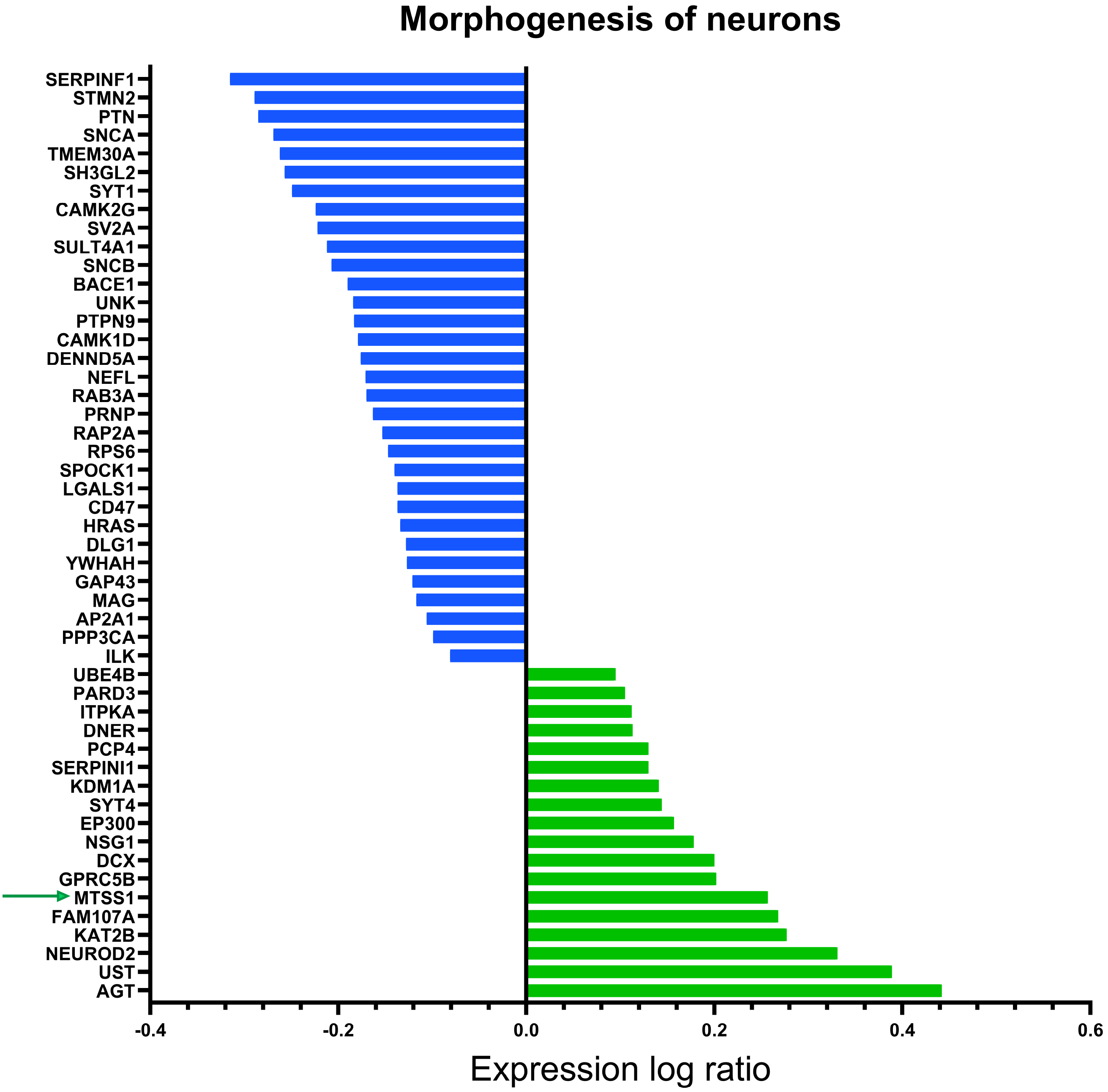
Morphogenesis of neurons
SERPINF1
STMN2
PTN
SNCA
TMEM30A
SH3GL2
SYT1
CAMK2G
SV2A
SULT4A1
SNCB
BACE1
UNK
PTPN9
CAMK1D
DENND5A
NEFL
RAB3A
PRNP
RAP2A
RPS6
SPOCK1
LGALS1
CD47
HRAS
DLG1
YWHAH
GAP43
MAG
AP2A1
PPP3CA
ILK
UBE4B
PARD3
ITPKA
DNER
PCP4
SERPINI1
KDM1A
SYT4
EP300
NSG1
DCX
GPRC5B
MTSS1
FAM107A
KAT2B
NEUROD2
UST
AGT
-0.4
-0.2
0.0
0.2
0.4
0.6
Expression log ratio

B

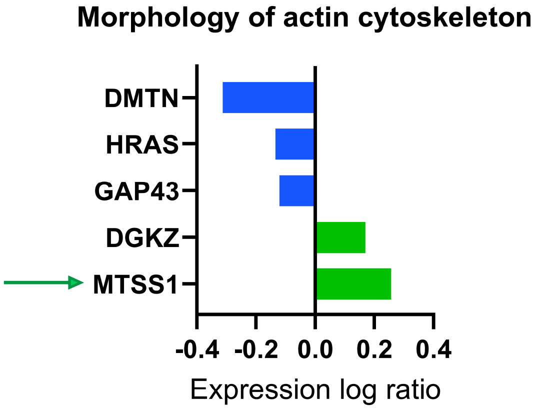
Morphology of actin cytoskeleton
DMTN
HRAS
GAP43
DGKZ
MTSS1
-0.4
-0.2
0.0
0.2
0.4
Expression log ratio

Figure S1. IPA pathway analysis of proteomics data.

IPA results from proteomics data comparing PKCγ-A24E+/+ versus PKCγ-A24Ewt/wt mice at three weeks-old show Mtss1 as one of the differentially enriched proteins involved in morphogenesis of neurons (**A**) and morphology of actin cytoskeleton (**B**). Blue proteins represent downregulation and green proteins represent upregulation. Green arrows point Mtss1 in both graphs.

Fig. S2

A

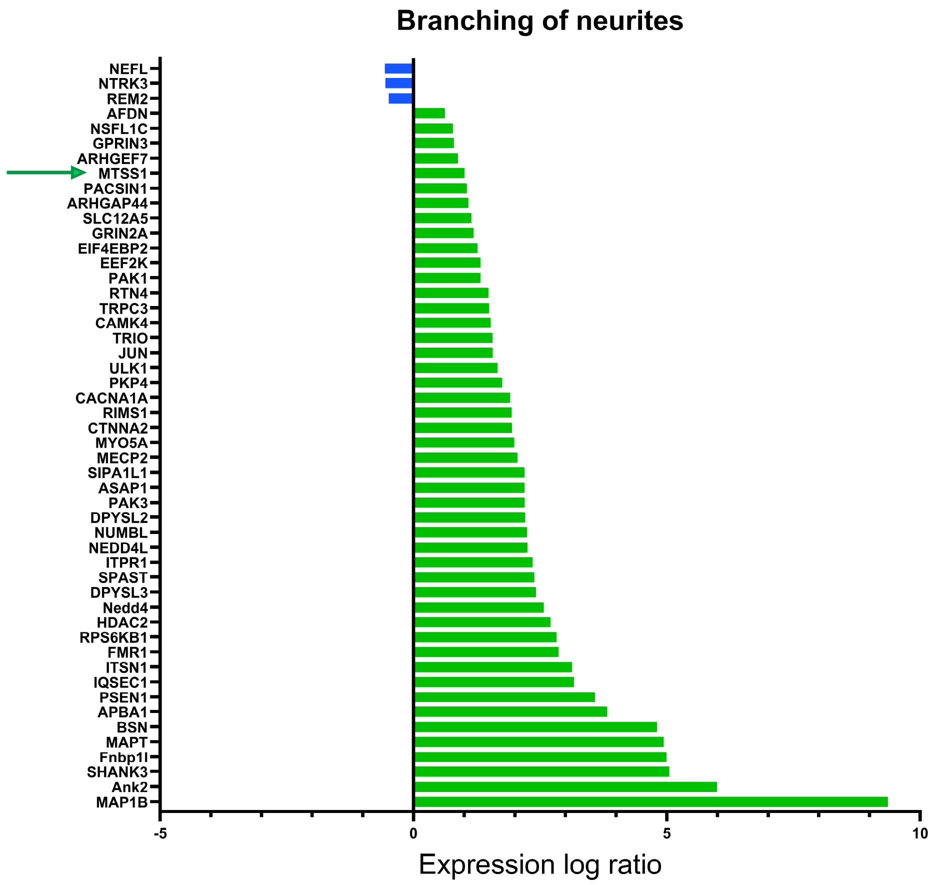

Branching of neurites
NEFL
NTRK3
REM2
AFDN
NSFL1C
GPRIN3
ARHGEF7
MTSS1
PACSIN1
ARHGAP44
SLC12A5
GRIN2A
EIF4EBP2
EEF2K
PAK1
RTN4
TRPC3
CAMK4
TRIO
JUN
ULK1
PKP4
CACNA1A
RIMS1
CTNNA2
MYO5A
MECP2
SIPA1L1
ASAP1
PAK3
DPYSL2
NUMBL
NEDD4L
ITPR1
SPAST
DPYSL3
Nedd4
HDAC2
RPS6KB1
FMR1
ITSN1
IQSEC1
PSEN1
APBA1
BSN
MAPT
Fnbp1l
SHANK3
Ank2
MAP1B
-5
0
5
10
Expression log ratio

B

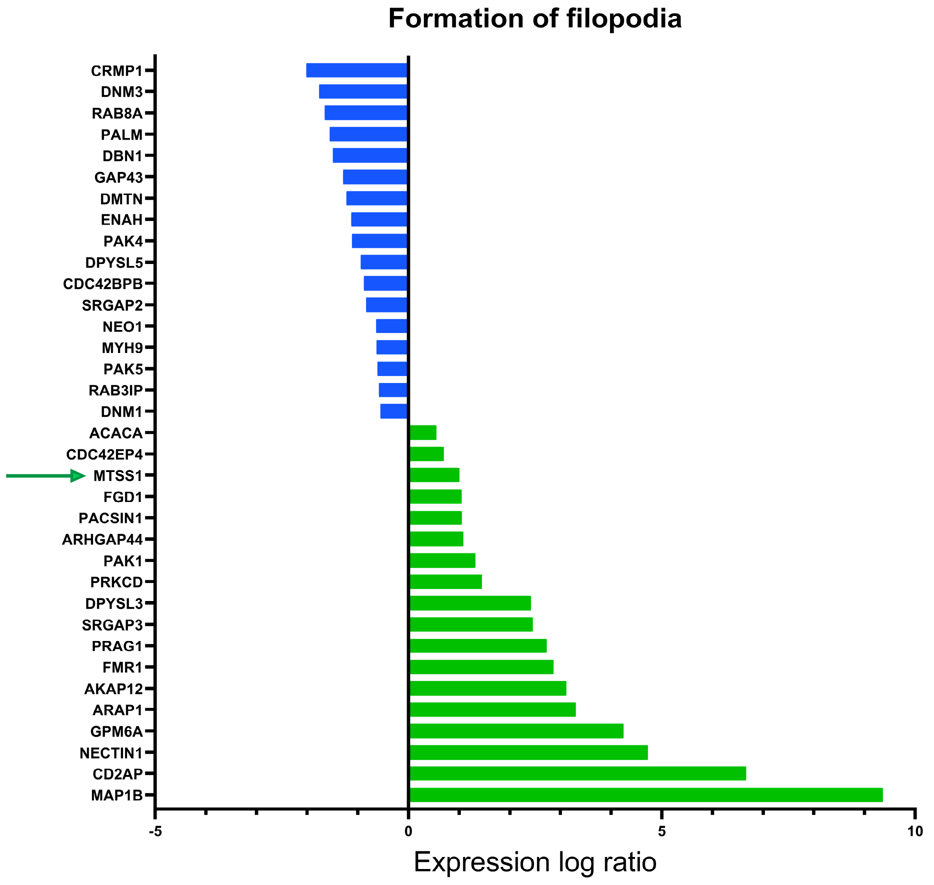

Formation of filopodia
CRMP1
DNM3
RAB8A
PALM
DBN1
GAP43
DMTN
ENAH
PAK4
DPYSL5
CDC42BPB
SRGAP2
NEO1
MYH9
PAK5
RAB3IP
DNM1
ACACA
CDC42EP4
MTSS1
FGD1
PACSIN1
ARHGAP44
PAK1
PRKCD
DPYSL3
SRGAP3
PRAG1
FMR1
AKAP12
ARAP1
GPM6A
NECTIN1
CD2AP
MAP1B
-5
0
5
10
Expression log ratio

Figure S2. IPA pathway analysis of phospho-proteomics data.

IPA results from phospho-proteomics data comparing PKCγ-A24E+/+ versus PKCγ-A24Ewt/wt mice at three weeks-old show that many proteins with an altered phosphorylation are involved in branching of neurites (**A**) and formation of filopodia (**B**), including Mtss1. Blue proteins represent a decrease in phosphorylation and green proteins represent an increase. Green arrows point Mtss1 in both graphs.

Fig. S3

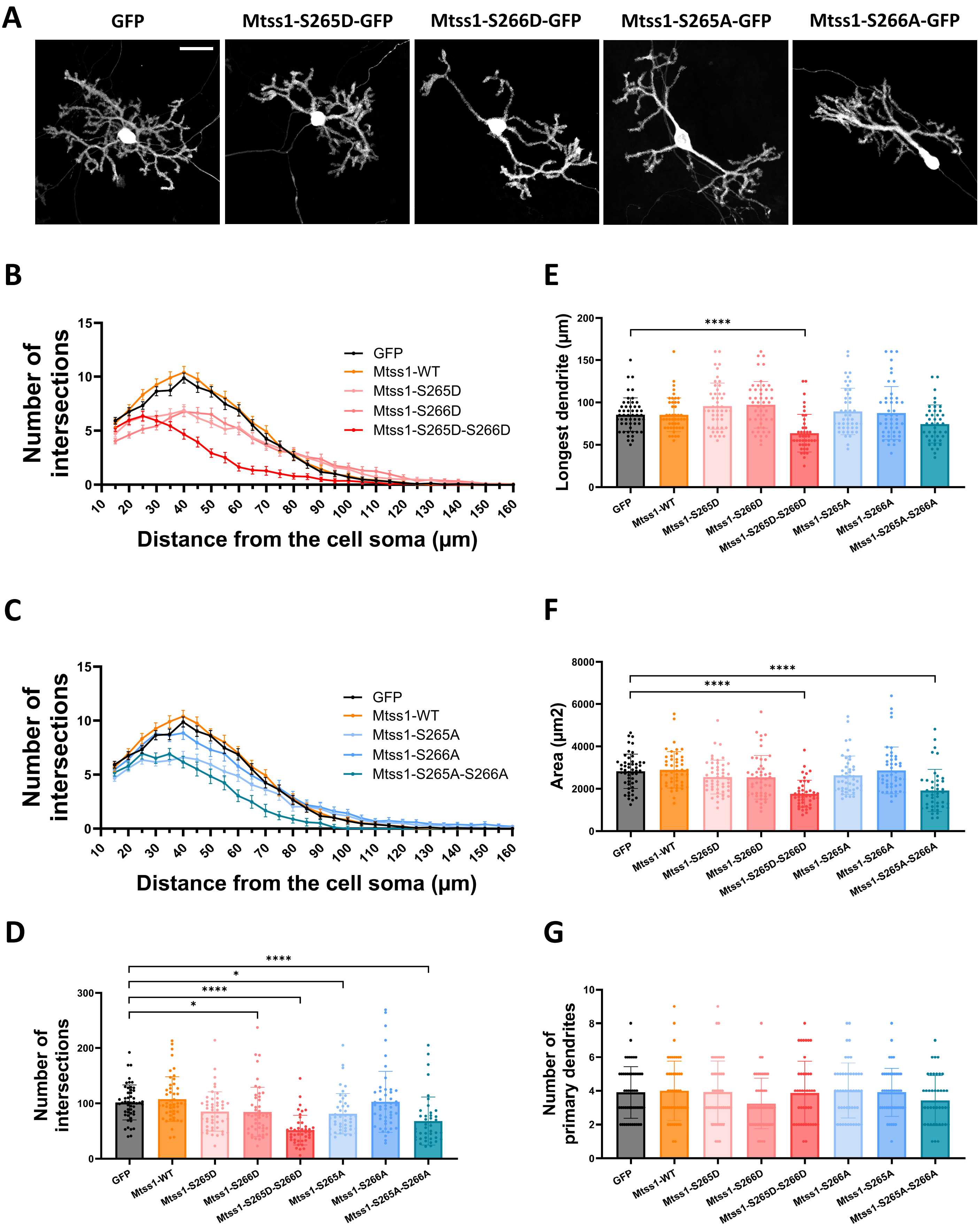

A
GFP
Mtss1-S265D-GFP
Mtss1-S266D-GFP
Mtss1-S265A-GFP
Mtss1-S266A-GFP
B
Number of intersections
GFP
Mtss1-WT
Mtss1-S265D
Mtss1-S266D
Mtss1-S265D-S266D
Distance from the cell soma (µm)
C
Number of intersections
GFP
Mtss1-WT
Mtss1-S265A
Mtss1-S266A
Mtss1-S265A-S266A
Distance from the cell soma (µm)
D
Number of intersections
****
*
****
*
GFP
Mtss1-WT
Mtss1-S265D
Mtss1-S266D
Mtss1-S265D-S266D
Mtss1-S265A
Mtss1-S266A
Mtss1-S265A-S266A
E
Longest dendrite (µm)
****
F
Area (µm2)
****
****
G
Number of primary dendrites

Figure S3. Single phospho-mimetic and phospho-defective mutations in Mtss1-S265 and Mtss1-S266 are not sufficient to trigger a dendritic disruption.

(**A**) Representative images of GFP-control, Mtss1-S265D-GFP, Mtss1-S266D-GFP, Mtss1-S265A-GFP, and Mtss1-S266A-GFP transfected PCs in DCCs at DIV21. Fluorescence detection: GFP. Scale bar = 20 µm. (**B**) Sholl analysis of phospho-mimetic and (**C**) phospho-defective single mutations are displayed in two different plots, each including the GFP-control, Mtss1-WT-GFP, and the corresponding double phospho-mimetic or double phospho-defective mutations. Data represented as mean ± SEM. (**D**) Total number of intersections, (**E**) area, (**F**) longest dendrite, and (**G**) number of primary dendrites of PCs transfected with GFP (n=52 cells), Mtss1-WT (n=48 cells), Mtss1-S265D (n= 48 cells), Mtss1-S266D (n= 49 cells), Mtss1-S265D-S266D (n=43 cells), Mtss1-S265A (n=45 cells), Mtss1-S266A (n=48 cells), and Mtss1-S265A-S266A (n=42 cells). Kruskal-Wallis test; * = p-value < 0,05; **** = p-value < 0,0001. Error bars (**D-G**) indicate SD. Data produced in 3-5 independent cultures per condition.

Figure S4. PC morphology changes at DIV10.

(**A**) Sholl analysis (data represented as mean ± SEM), (**B**) total number of intersections, (**C**) area, (**D**) longest dendrite, and (**E**) primary dendrites of PCs at DIV10, comparing PCs transfected with GFP (n=50 cells), Mtss1-WT (n=52 cells), Mtss1-S265D-S266D (n=57 cells), Mtss1-S265A-S266A (n=51 cells), and PKCγ-A24E (n=45 cells). Brown-Forsythe and Welch ANOVA test and Kruskal-Wallis test; * = p-value < 0,05; ** = p-value < 0,01; *** = P-value <0,001; **** = p-value < 0,0001. Error bars (**B-E**) indicate SD. Data produced in 3-5 independent cultures per condition.

Fig. S5

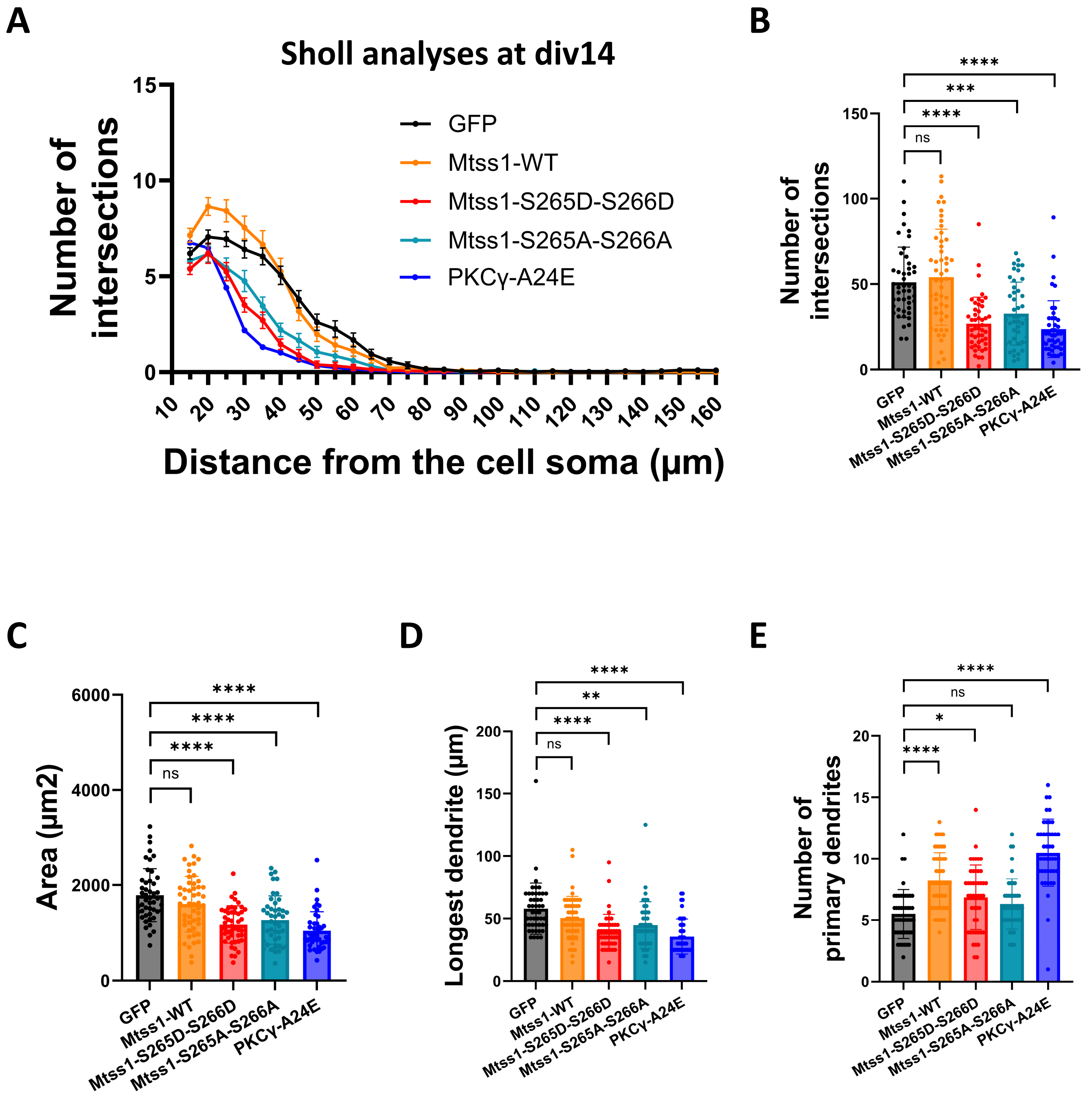
A
Sholl analyses at div14
Number of intersections
Distance from the cell soma (µm)
GFP
Mtss1-WT
Mtss1-S265D-S266D
Mtss1-S265A-S266A
PKCγ-A24E
B
Number of intersections
ns
****
***
****
C
Area (µm2)
ns
****
****
****
D
Longest dendrite (µm)
ns
****
**
****
E
Number of primary dendrites
****
*
ns
****

Figure S5. PC morphology changes at DIV14.

(**A**) Sholl analysis (data represented as mean ± SEM), (**B**) total number of intersections, (**C**) area, (**D**) longest dendrite, and (**E**) primary dendrites of PCs at DIV14, comparing PCs transfected with GFP (n=47 cells), Mtss1-WT (n=50 cells), Mtss1-S265D-S266D (n=46 cells), Mtss1-S265A-S266A (n=44 cells), and PKCγ-A24E (n=44 cells). Brown-Forsythe and Welch ANOVA test and Kruskal-Wallis test; * = p-value < 0,05; ** = p-value < 0,01; *** = P-value <0,001; **** = p-value < 0,0001. Error bars (**B-E**) indicate SD. Data produced in 3-5 independent cultures per condition.

Fig. S6

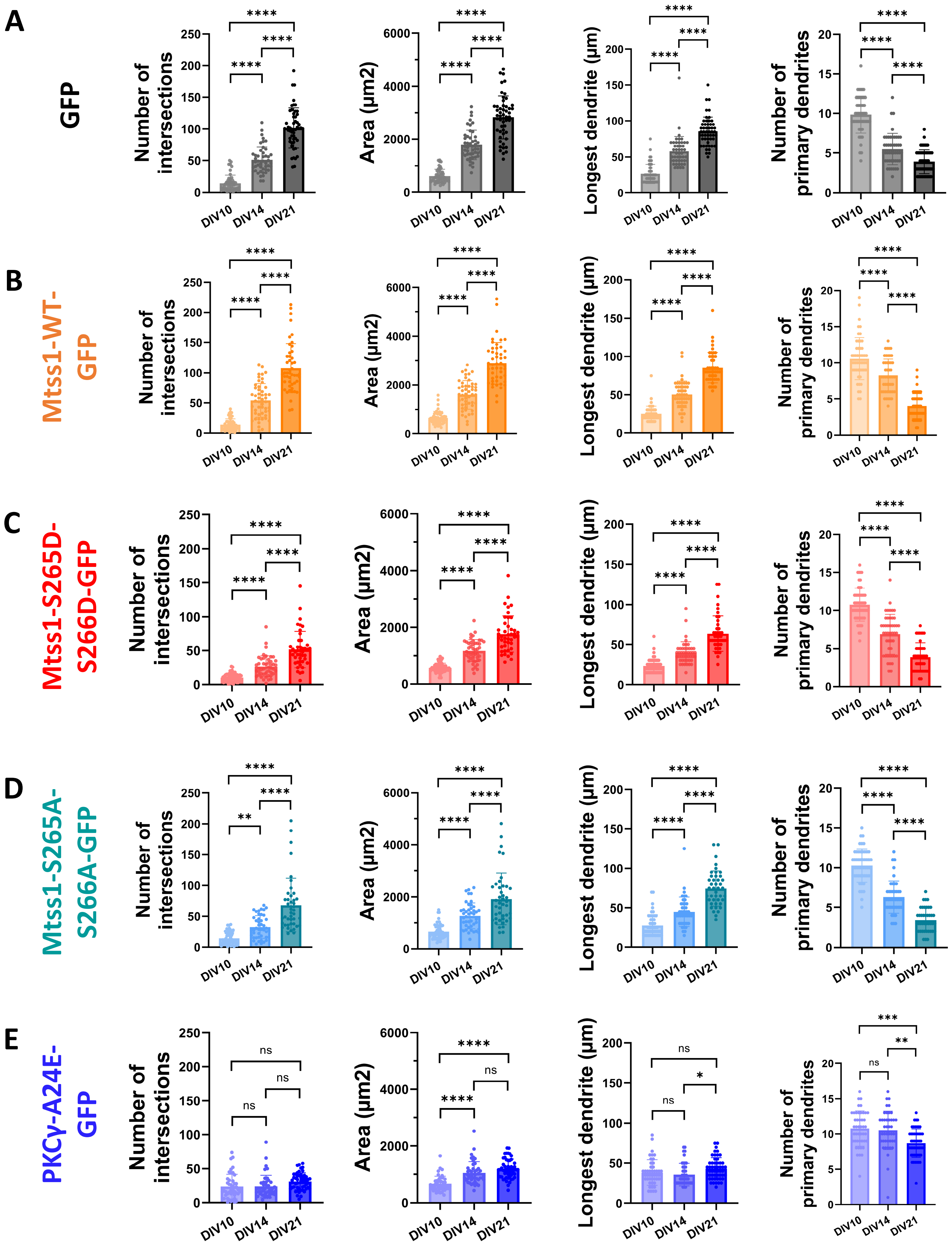
A
GFP
B
Mtss1-WT-GFP
C
Mtss1-S265D-S266D-GFP
D
Mtss1-S265A-S266A-GFP
E
PKCγ-A24E-GFP
Number of intersections
Area (μm2)
Longest dendrite (μm)
Number of primary dendrites
DIV10
DIV14
DIV21
****
***
**
*
ns

Figure S6. PKCγ-A24E impairs PC dendritic development at the stellate stage, whereas Mtss1 phosphorylation disrupts dendritic branch extension while maintaining developmental stages similar to GFP control PCs.

Quantification of the number of intersections, area, longest dendrite, and number of primary dendrites in PCs transfected with (**A**) GFP, (**B**) Mtss1-WT-GFP, (**C**) Mtss1-S265D-S266D-GFP, (**D**) Mtss1-S265A-S266A-GFP, and (**E**) PKCγ-A24E-GFP constructs, comparing DIV10, DIV14, and DIV21. Sample sizes: GFP DIV10 n = 50 cells, DIV14 n = 47 cells, DIV21 n = 52 cells; Mtss1-WT DIV10 n = 52 cells, DIV14 n = 50 cells, DIV21 n = 48 cells; Mtss1 S265D-S266D DIV10 n = 57 cells, DIV14 n = 46 cells, DIV21 n = 43 cells; Mtss1-S265A-S266A DIV10 n = 51 cells, DIV14 n = 44 cells, DIV21 n = 42 cells; PKCγ-A24E DIV10 n = 45 cells, DIV14 n = 44 cells, DIV21 n = 51 cells. One-way ANOVA; * = p-value < 0,05; ** = p-value < 0,01; *** = P-value <0,001; **** = P-value <0,0001. Error bars indicate SD. Data represent 3-5 independent cultures per condition.

Fig. S7

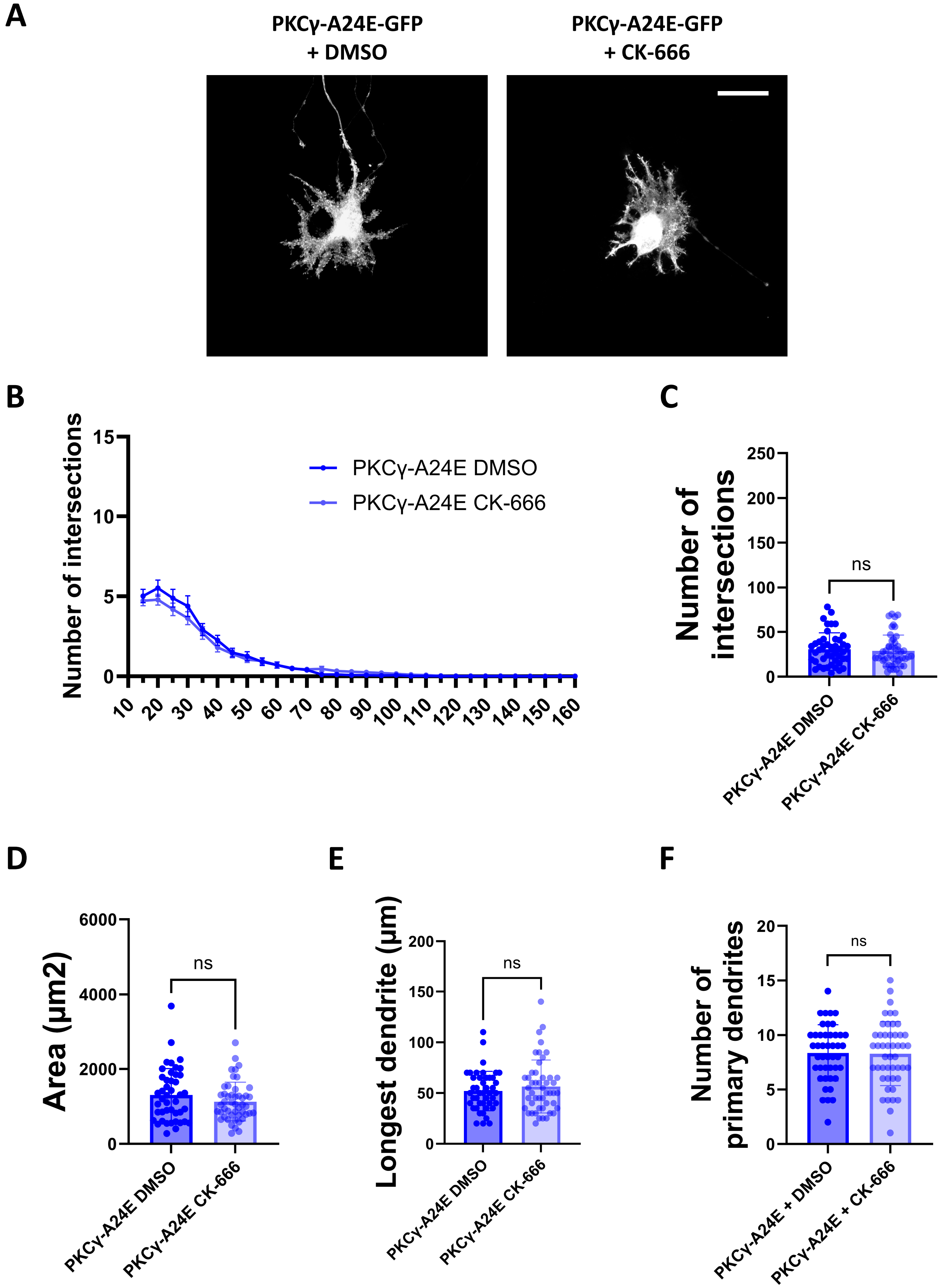
A
PKCγ-A24E-GFP + DMSO
PKCγ-A24E-GFP + CK-666
B
Number of intersections
PKCγ-A24E DMSO
PKCγ-A24E CK-666
C
Number of intersections
ns
PKCγ-A24E DMSO
PKCγ-A24E CK-666
D
Area (µm2)
ns
PKCγ-A24E DMSO
PKCγ-A24E CK-666
E
Longest dendrite (µm)
ns
PKCγ-A24E DMSO
PKCγ-A24E CK-666
F
Number of primary dendrites
ns
PKCγ-A24E + DMSO
PKCγ-A24E + CK-666

Figure S7. Dendritic disruption of PCs transfected with PKCγ-A24E cannot be rescued by CK-666 treatment.

(**A**) Representative images of PCs at DIV21, transfected with PKCγ-A24E-GFP, and biochemically treated with DMSO (Control) or CK-666 in DCCs. Fluorescence detection: GFP. Scale bar = 20 µm. (**B**) Sholl analysis (data represented as mean ± SEM), (**C**) total number of intersections, (**D**) area, (**E**) longest dendrite, and (**F**) number of primary dendrites of PCs transfected with PKCγ-A24E + DMSO (n=44 cells), and PKCγ-A24E + CK-666 (n=48 cells). Unpaired T-test. ns= non-significant. Error bars (**C-F**) indicate SD. Data produced in 3-5 independent cultures per condition.
