# Supplementary material for: PKCγ-mediated Phosphorylation of Mtss1 Regulates the Dendritic Outgrowth and Spine Development of Cerebellar Purkinje Cells
Source: Mol Neurobiol. 2025 Nov 25;63(1):168. doi: 10.1007/s12035-025-05526-9 (PMC12647342; doi:10.1007/s12035-025-05526-9)
Supplement: Supplementary file 2 — Supplementary file2 (PDF 2715 KB) [file 12035_2025_5526_MOESM2_ESM.pdf]

Supplementary material 2

Fig. S8

A

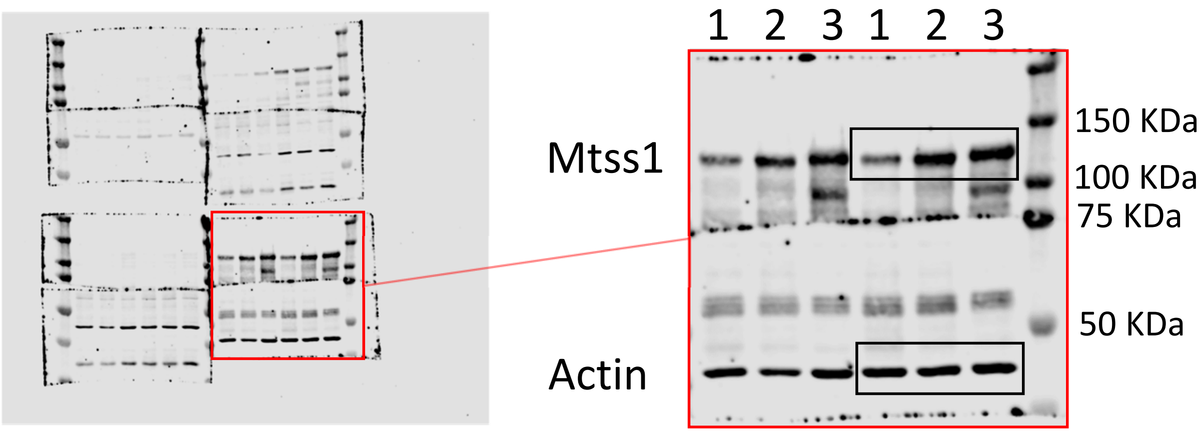

B

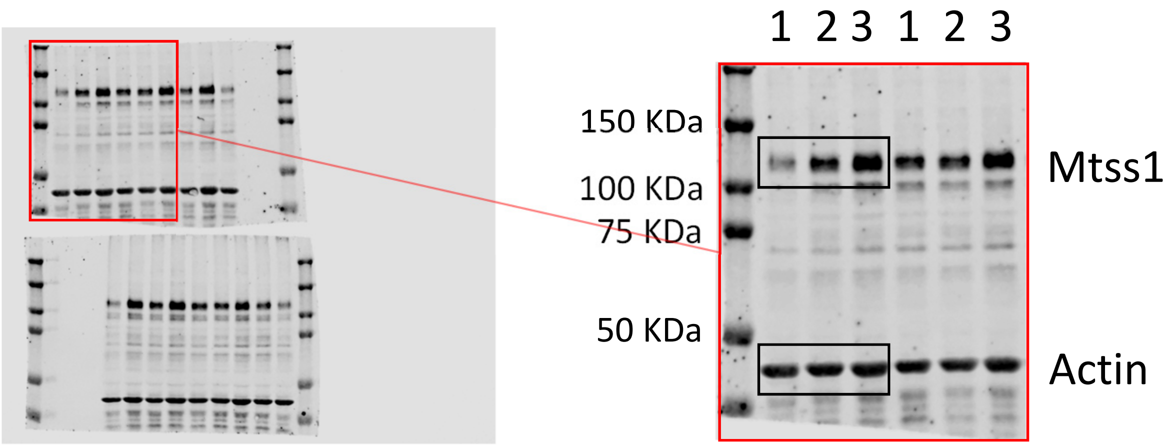

C

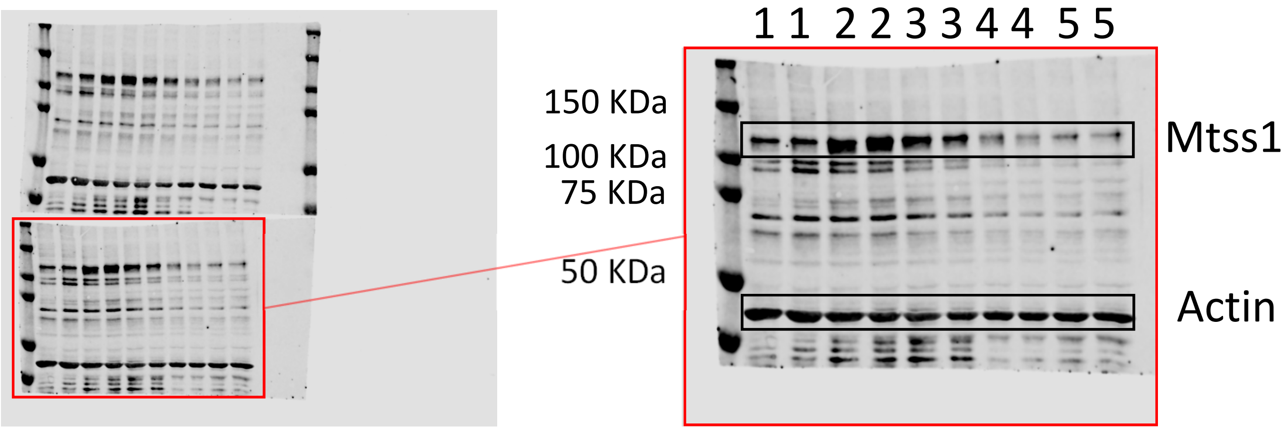

Figure S8. Original western blots from Figure 1.

(**A**) Samples: 1- Control, 2- PMA, 3- PKCγ-A24E. (**B**) Samples: 1- A24Ewt/wt, 2- A24Ewt/+, 3- A24E+/+. (C) Samples: 1- P7, 2- P14, 3- P21, 4- P35, 5- P266. The repeated samples show biological replicates. Squares mark the used bands for the manuscript.

Fig. S9

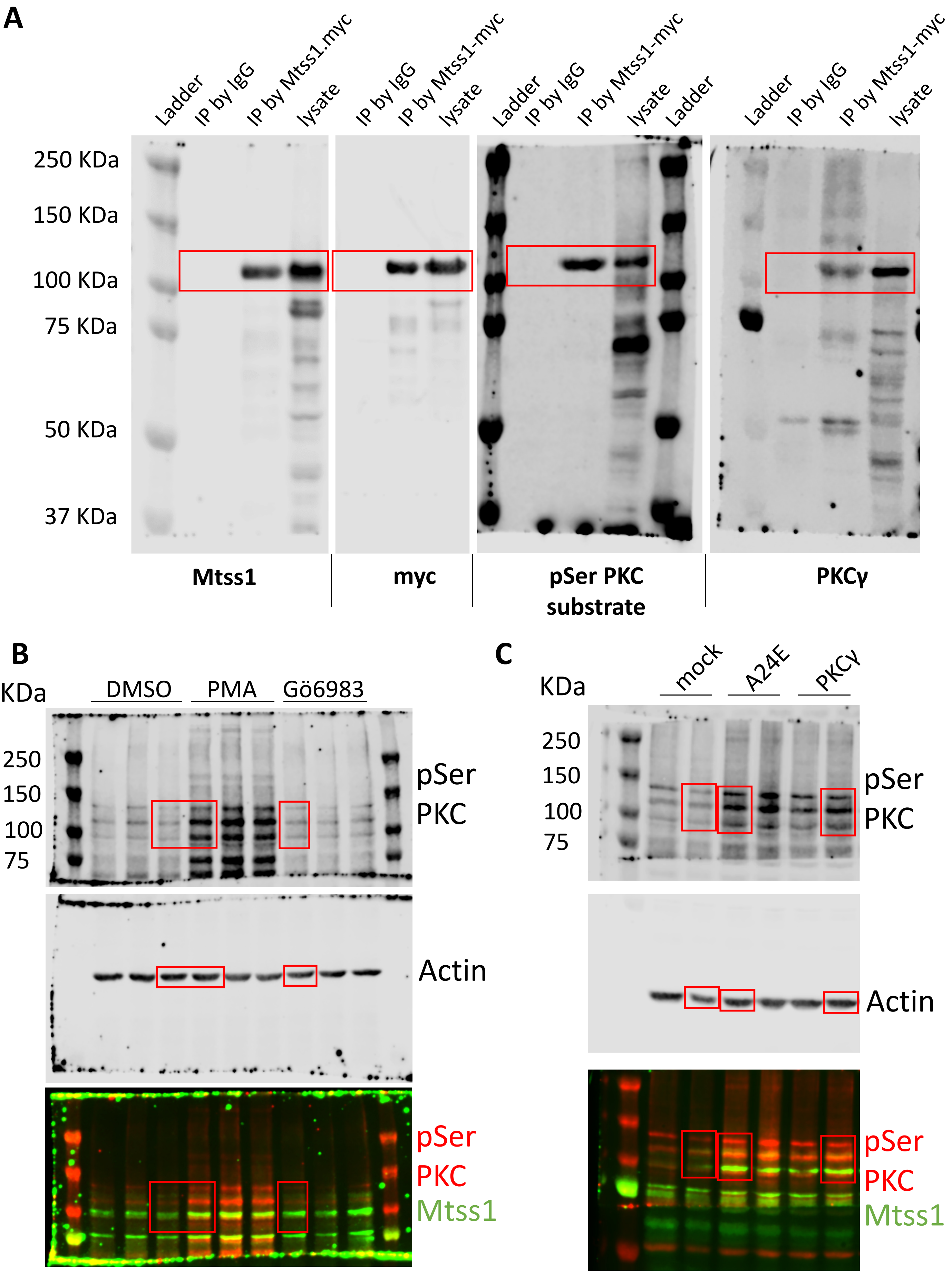
A
Ladder
IP by IgG
IP by Mtss1.myc
lysate
IP by IgG
IP by Mtss1-myc
lysate
Ladder
IP by IgG
IP by Mtss1-myc
lysate
Ladder
Ladder
IP by IgG
IP by Mtss1-myc
lysate
250 KDa
150 KDa
100 KDa
75 KDa
50 KDa
37 KDa
Mtss1
myc
pSer PKC substrate
PKCγ
B
KDa
DMSO
PMA
Gö6983
250
150
100
75
pSer PKC
Actin
pSer PKC
Mtss1
C
KDa
mock
A24E
PKCγ
250
150
100
75
pSer PKC
Actin
pSer PKC
Mtss1

Figure S9. Original western blots from Figure 2.

(**A**) Immunoblots stained with anti-Mtss1, anti-myc, anti-pSer PKC substrates, and anti-PKCγ, respectively, to show the IP results. (**B**) Western blot images with DMSO, PMA, and Gö6983 samples. (**C**) Western blot images with mock, PKCγ-A24E, and PKCγ-WT samples. The repeated samples show biological replicates. Squares mark the used bands for the manuscript.
